# Assessment of tick populations associated with capybaras in natural reserves and human-modified environments with or without invasive plants in the state of São Paulo, Brazil

**DOI:** 10.1007/s10493-026-01127-w

**Published:** 2026-03-14

**Authors:** Matheus Pasini-Martins, Rubia Diaz Taveiros Kuhne, Carolina Moura de Oliveira, Lina C. Binder, Igor S. Silito, Adriano Pinter, Fernanda B. Passos Nunes, Thiago F. Martins, José Henrique de Hildebrand e Grisi Filho, Wayne Dawson, Philip A. Stephens, Dalva Maria da Silva Matos, Marcelo B. Labruna

**Affiliations:** 1 Department of Preventive Veterinary Medicine and Animal Health, Faculty of Veterinary Medicine and Animal Science, University of São Paulo, São Paulo, SP Brazil; 2 Department of Evolution, Ecology and Behaviour, Institute of Infection, Veterinary and Ecological Sciences, University of Liverpool, Crown Street, Liverpool, L69 7ZB UK; 3 Conservation Ecology Group, Department of Biosciences, Durham University, South Road, Durham, DH1 3LE UK; 4 Departament of Hydrobiology, Universidade Federal de São Carlo, São Carlos, SP Brazil

**Keywords:** Amblyomma, Hydrochoerus hydrochaeris, Hedychium coronarium, Megathyrsus maximus, Cenchrus purpureus

## Abstract

In southeastern Brazil, capybaras (*Hydrochoerus hydrochaeris*) are main hosts of the ticks *Amblyomma dubitatum* and *Amblyomma sculptum*; the latter has great medical importance for being the main vector of *Rickettsia rickettsii*, the agent of Brazilian spotted fever (BSF). This study aimed to quantify ticks in areas invaded and not invaded by exotic plants (*Hedychium coronarium*,* Cenchrus purpureus* or *Megathyrsus maximus*) within capybaras’ living areas in São Paulo state. Tick populations were quantified in 24 capybara habitats, eight in natural reserves and 16 human-modified landscapes; the latter were composed of eight BSF-endemic areas and eight BSF-nonendemic areas. In the 24 areas, ticks were sampled in plots of natural forests and plots with at least one of the three invasive plants. Both presence and density of *A. sculptum* were higher in human-modified landscapes, while the presence and density of *A. dubitatum* tended to be similar among human-modified landscapes and natural reserve areas. The two tick species were collected both in areas colonized by invasive plants (*H. coronarium*, *M. maximus*, and/or *C. purpureus*) and in areas of non-invaded vegetation. However, a higher density of *A. dubitatum* was observed in areas of *H. coronarium* and a lower density of *A. sculptum* in this type of vegetation. Within human-modified landscapes, there was a higher density of *A. sculptum* in BSF-endemic areas when compared to non-endemic areas, unlike *A. dubitatum*, which remained at an almost constant density and presence among BSF-endemic and non-endemic areas. Plant invasions may have some role in the ecology of capybara-associated ticks.

**Supplementary Information:**

The online version contains supplementary material available at 10.1007/s10493-026-01127-w.

## Introduction

The introduction of exotic species has been shown to have a direct impact on the local reduction of species richness. Invasive macrophytes can have a huge ecological impact on native communities and ecosystems. One impact that has not been studied much is how they change the behavior of herbivorous mammals that might use the invaded areas (Stewart et al. 2021). These changes might arise as a result of the way the invading plants alter vegetation quality or structure, with knock-on effects on the preferences of herbivores, or their use of the area for rest or escape. In Brazil, especially in the state of São Paulo, the invasive *Hedychium coronarium* (white ginger lily), *Megathyrsus maximus* (Guinea grass) and *Cenchrus purpureus* (elephant grass) are invasive herbaceous plants with a high capacity for dispersal (Zenni and Ziller 2011). These species are native to Africa (*Cenchrus* and *Megathyrsus*) and Asia (*Hedychium*), and are now widespread in many parts of the country (Instituto Horus 2025). *Hedychium coronarium*, in particular, forms large vegetative aggregates in littoral areas and swampy environments, altering the vegetation structure in these regions. This macrophyte also causes changes in soil water percolation and evapotranspiration, especially when found forming monodominant stands (Vergne et al. 2023).

The capybara (*Hydrochoerus hydrochaeris*) is the world’s largest extant rodent species, weighing on average ≈ 60 kg, and is native to all South American countries except Chile (Moreira et al. 2013). Capybaras are semiaquatic grazers that live in social groups of typically 10 to 40 individuals, although larger groups of up to 100 individuals have been reported (Moreira et al. 2013). Due to their semiaquatic habits, groups of capybaras always live near a body of water, but they consume foliage of various plants in nearby vegetation, such as grasses, shrubs, and agricultural crops (Moreira et al. 2013). Exotic grasses such as *M. maximus* and *C. purpureus* are included in the list of foraging grasses grazed by capybaras (Moreira et al. 2013). While the grazing of *H. coronarium* by capybaras is still under debate, the association of this exotic plant species with aquatic environments makes it abundant in many living areas of capybaras in Southeastern Brazil (Castro et al. 2013).

In southeastern Brazil, capybaras act as main hosts of all active stages of two tick species, *Amblyomma dubitatum* and *Amblyomma sculptum*, which are usually found coinfesting capybaras (Luz et al. 2019). *Amblyomma dubitatum* has been reported as the true capybara tick, since all known populations of this tick species are primarily related to the presence of the capybara, its main host species (Nava et al. 2010). The primary association of large populations of *A. sculptum* with capybaras seems to have occurred more recently, due to anthropogenic factors that altered both the environment and the behavior of capybaras in human-modified landscapes (Luz et al. 2019; Dias et al. 2020; Lopes et al. 2021). Indeed, *A. sculptum* is medically important in Brazil, where it is the main vector of *Rickettsia rickettsii*, the bacterium that causes Brazilian spotted fever (BSF) — the deadliest tick-borne disease affecting humans in the Western Hemisphere (Foley et al. 2025). In addition to being the main hosts of *A. sculptum* in BSF-endemic areas, capybaras also act as infection source (amplifying host) of *R. rickettsii* to ticks (Labruna et al. 2013, Ramírez-Hernández et al. 2020). Due to this close relationship between *A. sculptum*, *R. rickettsii*, and capybaras, the reemergence of BSF in southeastern Brazil has been associated with the expansion of capybaras in this region since the end of the last century (Labruna 2013, Bovo et al. 2016).

Besides being strictly hematophagous parasites, ticks depend on specific microenvironmental characteristics to undergo off-host developmental stages, such as molting from larvae to nymphs and nymphs to adults, and female oviposition and egg incubation periods (Sonenshine 1991). Therefore, there is a primary dependency on environmental characteristics for the establishment of tick populations. A change in vegetation caused by introduced and invasive plant species could alter the microenvironmental characteristics necessary for tick development. For instance, *M. maximus* and *C. purpureus* are grass species that create microclimate with relatively high humidity near the soil surface and abundant vertical structures for host-questing, conditions that are particularly suitable for *A. sculptum*, which relies on vegetation architecture to maximize contact with passing hosts (Pajuaba Neto et al. 2018). Moreover, the frequent use of these grass-dominated areas by capybaras likely enhances the local deposition of engorged ticks and the subsequent development of immature stages, reinforcing tick population maintenance in these habitats (Ramos et al. 2019).

Thus, this study aimed to quantify ticks (*A. dubitatum* and *A. sculptum*) in areas invaded and not invaded by exotic plants within the habitat of capybaras in the state of São Paulo, based on the hypothesis that areas dominated by invaded plants could be more favorable for ticks. To this end, tick populations were quantified in capybara habitats in both natural reserves and human-modified landscapes, provided that the living areas of capybaras included plots of natural riparian forests and plots with at least one of the three invasive plants: *H. coronarium*, *M. maximus* and/or *C. purpureus.*

## Materials and methods

### Study areas

**Fig. 1:**
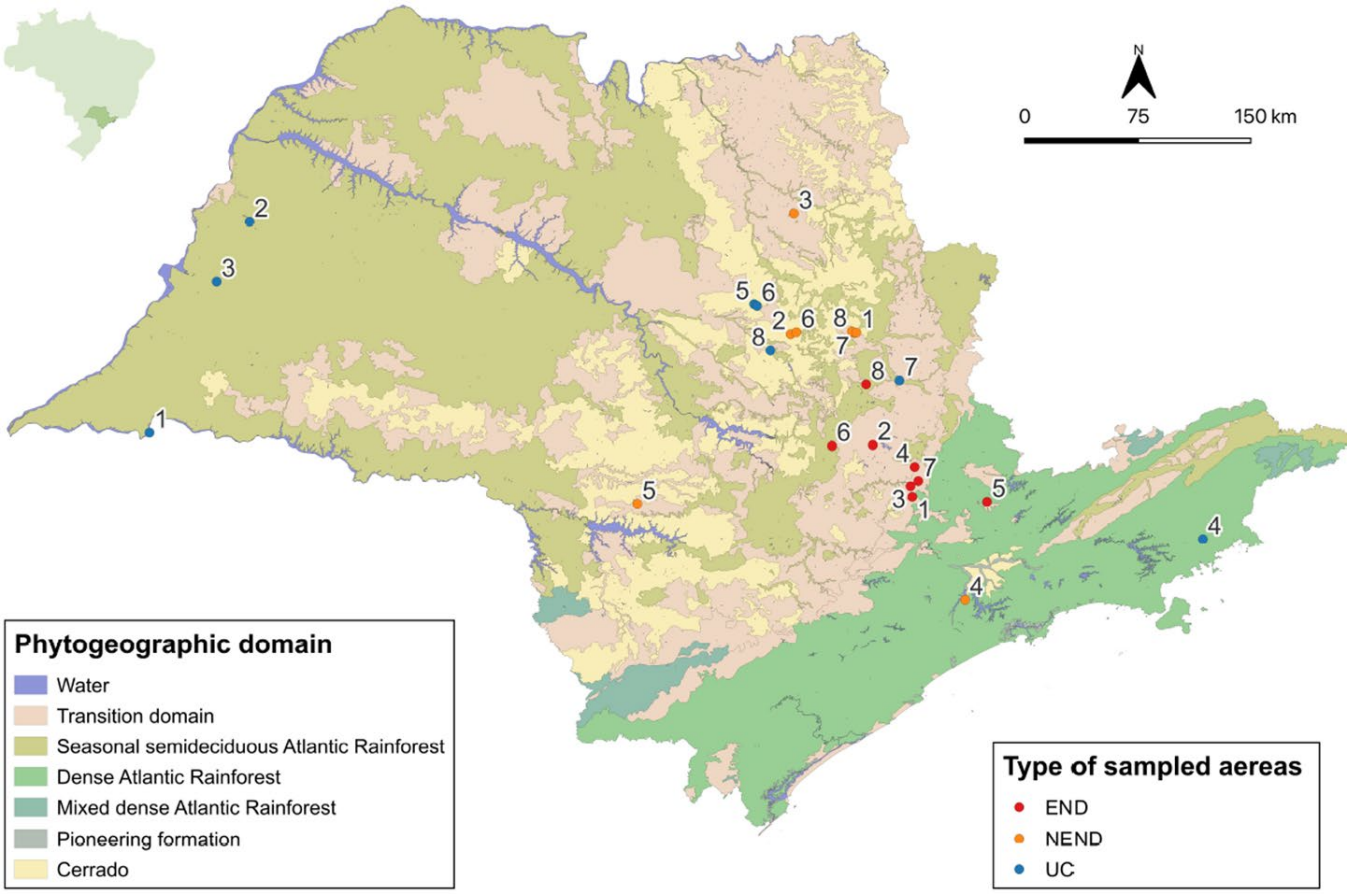
Geographical locations of the 24 areas sampled in the state of São Paulo during autumn/winter of 2023 or 2024. END1 to END8 consisted of human-modified landscape areas endemic for Brazilian spotted fever; NEND1 to NEND8 consisted of human-modified landscape areas not endemic for Brazilian spotted fever; and UC1 to UC8 consisted of natural reserve areas within conservation units of the Atlantic Rainforest biome

This study covered 24 areas, each with an established population of capybaras. These 24 areas were divided into three categories, each of them containing eight areas (Table 1). Two categories consisted of human-modified landscapes near urban areas, whereas a third category consisted of natural reserves within conservation units of the Atlantic Rainforest biome. Among the human-modified landscapes, eight areas (designated as END1 to END8) were endemic for BSF, with recent circulation of *R. rickettsii* based on confirmed clinical cases of BSF or the demonstration of *R. rickettsii* previous infection in capybaras through serosurveys. By contrast, the other eight human-modified landscape areas (designated as NEND1 to NEND8) were not endemic for BSF, as demonstrated by serosurvey studies on capybaras or horses, which indicated absence of *R. rickettsii* previous infection or exposure in these animals. The classification of these END and NEND areas as endemic and nonendemic for BSF, based on serosurvey of capybaras or horses, followed the criteria of the official guidelines of the State of São Paulo for classification of BSF-endemic areas (São 2023). END and NEND areas included university campuses, public parks, and residential parks. Finally, the eight natural reserves (designated as UC 1 to UC8) consisted of natural preservation areas within Conservation Units in the state of São Paulo. The 24 areas are geographically indicated in Fig. 1. Our purpose in sampling these three categories of areas was because a previous study in capybara living areas found higher tick abundance in human-modified landscapes than in natural areas; in addition, among the human-modified landscapes, it was shown that the tick density (especially *A. sculptum*) was significantly higher in BSF-endemic areas than in non-endemic areas (Luz et al. 2019). Therefore, our intention to include these three categories of areas in our study was also to verify if these previously reported differences could be related to the exotic plants here investigated.

**Table 1:** Data of the 24 visited areas of the state of São Paulo, in which ticks were collected in the present study. Eight areas (END1 to END8) consisted of human-modified landscapes endemic for Brazilian spotted fever; eight areas (NEND1 to NEND8 consisted of human-modified landscapes not endemic for Brazilian spotted fever; and eight areas (UC1 to UC8) consisted of natural reserves within Conservation Units. In each area, ticks were sampled in non-invaded sites (N.i.s.), which were locations dominated by native vegetation without exotic grass and *Hedychium coronarium.* When present, locations dominated by *H. coronarium* (H. cor), and locations dominated by any of the grasses *Cenchrus purpureus* and *Megathyrsus maximus* (C.p/M.m) were also sampled

Area code	Municipality	Date of visit	Name of the area: general description	BSF status	Sampled plots (75 cm^2^/area)		
					H. cor	C.*p*/M.m	*N*.i.s.
END1	Itupeva	May 2023	Residential Park: area containing artificial lakes and cultivated grasses amid forest fragments.	Endemic^2^	yes	yes	yes
END2	Americana	Jun. 2024	Carioba sewage treatment station: area composed of abandoned grass fields and degraded riparian forests along the Piracicaba River.	Endemic^1^	no	yes	yes
END3	Campinas	Jun. 2024	Jardim Camélias: area composed of degraded riparian forests along the Capivari River.	Endemic^2^	no	yes	yes
END4	Campinas	Jun. 2024	Ribeirão das Pedras Public Park: area containing an artificial lake and cultivated grasses amid forest fragments.	Endemic^2^	no	yes	yes
END5	Atibaia	Jun. 2024	Residential Park: area containing artificial lakes and cultivated grasses amid forest fragments.	Endemic^2^	yes	no	yes
END6	Piracicaba	Jun. 2024	Airport Lake: area in the University of São Paulo “Esalq” campus, composed of abandoned grass fields and degraded riparian forests surrounding the Airport Lake.	Endemic^1^	no	yes	yes
END7	Campinas	Jul. 2024	São Vicente Public Park: area containing a lake irrigated by streams, surrounded by degraded forest fragments.	Endemic^2^	no	yes	yes
END8	Araras	Jul. 2024	UFSCAR Araras campus: area in the campus of Araras, composed of abandoned grass fields and degraded riparian forests surrounding the main lake.	Endemic^1^	no	yes	yes
NEND1	Pirassununga	May 2023	Risca-Faca Lake: area in the University of São Paulo campus Pirassununga, composed of corn crops interposed by livestock artificial pastures and degraded riparian forests along an artificial lake.	Non endemic^1^	yes	yes	yes
NEND2	São Carlos	May 2023	UFSCAR São Carlos campus: area in the Federal University of São Carlos campus of São Carlos, composed of abandoned grass fields and degraded riparian forests surrounding the main lake.	Non endemic^3^	yes	yes	yes
NEND3	Ribeirão Preto	Jun. 2023	University campus: area in the University of São Paulo campus Ribeirão Preto, composed of abandoned grass fields and degraded forests along two main streams.	Non endemic^1^	yes	yes	yes
NEND4	São Paulo	Jun. 2023	Residential Park: area containing riparian forest amid cultivated grass surrounding the Guarapiranga Dam.	Non endemic^3^	yes	no	yes
NEND5	Avaré	Sep. 2023	Florestal Park of Avaré: area composed of abandoned grass fields and degraded and preserved riparian forests surrounding the Main Lake and tributary streams.	Non endemic^3^	yes	yes	yes
NEND6	São Carlos	May 2024	Experimental Farm (Embrapa Pecuária Sudeste): area composed mainly by livestock artificial pastures and degraded riparian forests surrounding the Main Lake.	Non endemic^3^	yes	no	yes
NEND7	Pirassununga	Jun. 2024	Aguapé Lake: area in the University of São Paulo Pirassununga campus, composed of degraded riparian forests along an artificial lake.	Non endemic^1^	no	yes	yes
NEND8	Pirassununga	Aug. 2024	Captação Lake forest: area in the University of São Paulo Pirassununga campus, composed of artificial pastures and degraded riparian forests along a dammed river stream.	Non endemic^1^	yes	yes	yes
UC1	Teodoro Sampaio	May 2023	Morro do Diabo Estate Park: preserved seasonal semideciduous forest of the Atlantic Forest Biome.	Unknown	no	yes	yes
UC2	Nova Independência	May 2023	Rio Aguapeí Estate Park: preserved seasonal semideciduous forest of the Atlantic Forest Biome.	Unknown	no	yes	yes
UC3	Presidente Venceslau	May 2023	Rio do Peixe State Park: preserved seasonal semideciduous forest of the Atlantic Forest Biome.	Unknown	no	yes	yes
UC4	S. Luiz Paraitinga	Jun. 2023	Serra do Mar State Park, Santa Virgínia: preserved dense forest of the Atlantic Forest Biome.	Unknown	yes	yes	yes
UC5	Araraquara	Jul. 2023	Municipal Nature Park of Basalto: preserved area of the Cerrado Biome.	Unknown	yes	no	yes
UC6	Araraquara	Jul. 2023	Municipal Nature Park of Basalto, “DAAE”: preserved area of the Cerrado Biome in the watershed station.	Unknown	yes	no	yes
UC7	Mogi-guaçu	May 2024	Mogi-Guaçu Ecological Station: transition domain of seasonal semideciduous rainforest and Cerrado Biomes.	Unknown	no	yes	yes
UC8	Brotas	Jul. 2023	Mata do Rio Jacaré Ecological Station: preserved semideciduous forests of the Atlantic Forest and Cerrado Biomes.	Unknown	yes	yes	yes

^1^ Luz et al. (2019); ^2^unpublished data from the São Paulo State Environmental Secretary; ^3^unpublished data based on serological analyses of capybaras or horses done at the Laboratory of Parasitic Diseases of the Faculty of Veterinary Medicine of the University of São Paulo

### Tick sampling

In all study areas, ticks were collected from the environment during the autumn and early winter months (May to July), the peak activity season for the larval stage of *A. sculptum* and *A. dubitatum* in the state of São Paulo (Luz et al. 2019). Tick samplings were performed in locations where capybara groups usually spent most of their time, i.e., daytime living areas. This was based on studies that monitored capybaras with GPS collars, which showed that in human-modified landscapes, tick populations are concentrated in the riparian forests in which capybaras are usually found during daytime, when they are less mobile (Luz et al. 2019; Dias et al. 2020).

The tick collection sites (plots; herein after) in each of the 24 areas included three types of vegetation composition: (i) non-invaded sites - locations dominated by native vegetation with no exotic grass and no *H. coronarium*; (ii) locations dominated by *H. coronarium*; and (iii) locations dominated by any of the grasses *M. maximus* or *C. purpureus.* While all 24 sampled areas presented non-invaded plots, not all areas presented both *H. coronarium* and *M. maximus/C. purpureus* plots. That said, 25 m-tracks were defined for each of these three types of vegetation composition. In all 24 sampled areas, we sampled three transects (total of 75 m) in non-invaded plots, and when present, three transects of *H. coronarium*, and three transects of *C. purpureus*/*M. maximus*.

Ticks sampling concentrated during the larval peak of activity because a group of larvae in the vegetation indirectly represents a favorable environment for egg laying by engorged females, as well as for egg incubation and maintenance of viable larvae in the environment, whether diapausing or not (Labruna et al. 2003). Since the egg stage is the only immobile and slowest developing stage, it is the most sensitive to environmental disturbances in the tick cycle (Labruna et al. 2003). Thus, the quantification of larvae serves as a parameter to indicate the environmental favorability for the tick cycle. The ticks were collected using a flannel drag, as this is much more effective than CO₂ traps for collecting *A. sculptum* and *A. dubitatum* larvae (Oliveira et al. 2000; Luz et al. 2019; Szabó et al. 2024). Since the flannel drag also serves to collect nymphs and adults, these two stages were also sampled and collected into plastic tubes containing ethanol, even though nymphs and adults are usually less numerous in the fall, and the flannel dragging is less effective for adults of *A. dubitatum* than adults of *A. sculptum* (Szabó et al. 2024).

A 1 m^2^ piece of white fabric was used for the flannel drag and was dragged along predefined transects 25 m long and checked every 5 m to remove groups of larvae attached to the flannel. Whenever a group of larvae (larval cluster) was detected on the flannel, it was immediately removed with a piece of transparent adhesive tape (4.5 cm x 10 cm), which was then attached to a piece of white sulfite paper. Thus, the unit collected is the larval cluster, as each one contains hundreds to thousands of larvae, but usually originates from a single engorged female. This protocol was based on that of Luz et al. (2019), who quantified *A. sculptum* and *A. dubitatum* larvae in capybara-living areas of the state of São Paulo.

### Laboratory analysis of ticks

In the laboratory, the sulfite paper was examined under a stereoscopic microscope, where the larvae from each cluster were identified as *A. sculptum* or *A. dubitatum* through side-by-side comparison with larvae of the two species from laboratory colonies, as previously validated (Brites-Neto et al. 2013). The collected nymphs and adults were individually identified at the species level, according to the existing literature for adults (Barros-Battesti et al. 2006; Martins et al. 2016) and nymphs (Martins et al. 2010). The morphological identification of some larval clusters in natural reserve areas was confirmed by molecular analysis. This procedure was adopted because, unlike in human-modified landscapes, where only *A. sculptum* and *A. dubitatum* larvae have been collected by flannel drag in capybara habitats (Brites-Neto et al. 2013; Luz et al. 2019), the higher biodiversity of the UC areas could result in the collection of larval clusters of additional tick species that could be associated with other large mammals (tapirs, peccaries) that use the capybara habitat. Thus, to confirm the identification of some larval clusters, DNA was extracted from larval pools by the boiling technique (Horta et al. 2005). This procedure was done with 12 larval pools (up to 20 specimens per pool) from larval clusters that were collected in areas UC1 to UC6, and UC8. Extracted DNA samples were tested by a polymerase chain reaction (PCR) protocol targeting a 460-bp fragment of the tick mitochondrial 16 S rRNA gene, as previously described (Mangold et al. 1998). PCR products of the expected size were purified with ExoSap (USB, Cleveland, OH, USA) and sequenced (bidirectional Sanger sequencing) on an automated ABI sequencer (Applied Biosystems/Thermo Fisher Scientific, ABI 3500 Genetic Analyzer, Foster City, CA, USA). The resulting sequences were submitted to BLAST analysis (www.ncbi.nlm.nih.gov/blast) to determine the closest identities in GenBank.

### Data analysis

Through the results of the taxonomic identifications, we were able to produce a database on the quantities of ticks (*A. sculptum* and *A. dubitatum*) in relation to the study areas, the presence of invasive species, and the type of area (END, NEND and UC). Due to the large number of 25 m-transects with no ticks (null cells), the quantities of ticks collected in each transect were transformed into a variable presence or absence of ticks. Thus, a generalized linear mixed model was adjusted with tick presence as the response variable and the other variables, such as tick species *(A. sculptum* and *A. dubitatum*), type of predominating vegetation (*H. coronarium*, *C. purpureus*/*M. maximus* and non-invaded sites), and type of area (END, NEND and UC) as predictive variables, including the collection site as a random factor. In addition to these analyses, a similar model used type of area (END, NEND and UC) instead of type of vegetation as a predictor. The R software (R Core Team, 2024 - Version 2024.09.1 + 394) and the “lme4” package (Bates et al. 2015) were used to create the models and to perform the analyses. The grasses *C. purpureus*/*M. maximus* were analyzed together as a “single vegetation type” because they are exotic foraging grasses grazed by capybaras (Moreira et al. 2013) and have several similar environmental requirements, such as they cannot tolerate flooded or poorly drained soils and do not grow in predominantly shaded areas (Paciullo et al. 2017; Heuzé et al. 2020; Bilgin 2021).

A second type of analysis was based on density of ticks. Since we used a 1 m^2^ white flannel for tick dragging, we assumed an area of 25 m^2^ that was sampled in each 25 m-long transect. Hence, the density of ticks in each plot was calculated by dividing the total number of collected ticks (larval cluster, nymphs or adults) by the dragged area, which was 75 m^2^ in each sampled vegetation plot [(three dragged transects of 25 m) x (a 1 m^2^ flannel) = 75 m^2^]. This new variable (density of ticks) was included as a response variable; type of area, type of vegetation, and life stage of the ticks were used as predictor variables. In this case, tick counts per sample unit provided a measure of frequency in the environment during the study period, provided that the sampling effort is recorded and the different exposures are properly standardized (Nielson and Sawyer 2013). In studies involving count variables, it is common practice to fit Poisson or Negative Binomial (NB) regression models. In the Poisson regression model, it is assumed that the variable of interest represents non-negative counts, and that its mean is equal to its variance: *E*[*Y*] = *µ* and *Var* (*Y*) = *µ.* However, in the present study, it was found that the variation in counts was greater than the mean, characterizing a case of overdispersion (Yang et al. 2007). Given this, we opted to use a Negative Binomial regression model, which is more appropriate because it allows the variance to exceed the mean; *Var* (*Y*) > *E*[*Y*] (Lee et al. 2012). Parameterization with a logarithmic link function was adopted, whose variance is expressed by:

$$Var\left({Y}_{i}\right)={\mu}_{i}+\frac{{\mu}_{i}^{2}}{\theta},$$

where *θ* represents the dispersion parameters. According to Hilbe (2011), high values of the dispersion parameter (*θ*) indicate a lower degree of overdispersion, and, at the limit where *θ* → ∞, the model approximates the Poisson model.

The following were included as predictor variables: vegetation type (*H. coronarium*, *C. purpureus*/*M. maximus* and non-invaded sites), the type of area (END, NEND and UC), the tick species (*A. sculptum* and *A. dubitatum*) and the stage of development (larva, nymph, adult), as well as the interactions necessary to capture changes in effect between species and environment. In this case, the interaction between the tick species and the area and between the tick species and the vegetation type was evaluated. This was done to assess whether the difference between tick species changes according to the environment, as well as to show where this difference actually occurs.

$$logE\left[{Y}_{i}\right]={\beta}_{0}+{x}_{i}{\beta}_{0}+log\left(areasize\right),$$

in which the term log (area size) was included as an offset in order to standardize exposure and enable the estimation of tick densities. The R software (R Core Team, 2024) and the MASS package (Venables and Ripley 2002) were used. A significance level of 5% was assumed, and the reference for comparison corresponded to the type of uninvaded vegetation (non-invaded sites) and the type of conservation unit (UC) area.

## Results

The 24 sampled areas were visited during the fall/winter seasons of 2023 and 2024. Among these, 22 were visited from mid fall to early winter: 12 areas between May and July of 2023, and 10 areas between May and July of 2024. Due to logistic constraints, two areas were visited only during mid to late winter – one in August 2023 and another in September 2024 (Table 1).

Among the three types of vegetation composition sampled for ticks, non-invaded sites (forest fragments without *H. coronarium* and *C. purpureus*/*M. maximus* grass) were sampled in all 24 areas. Because sites dominated by *H. coronarium* were not found in the capybara living places of some areas, this type of site was sampled in 13 areas (two END areas, seven NEND areas, and four UC areas). In parallel, sites dominated by *C. purpureus*/*M. maximus* grass were sampled in 19 areas (seven END areas, six NEND areas, and six UC areas); overall, eight areas had all three types of sites sampled for ticks (Table 1). A general aerial view of the 24 sampled areas is shown in Supplementary Fig. S1.

**Fig. 2:**
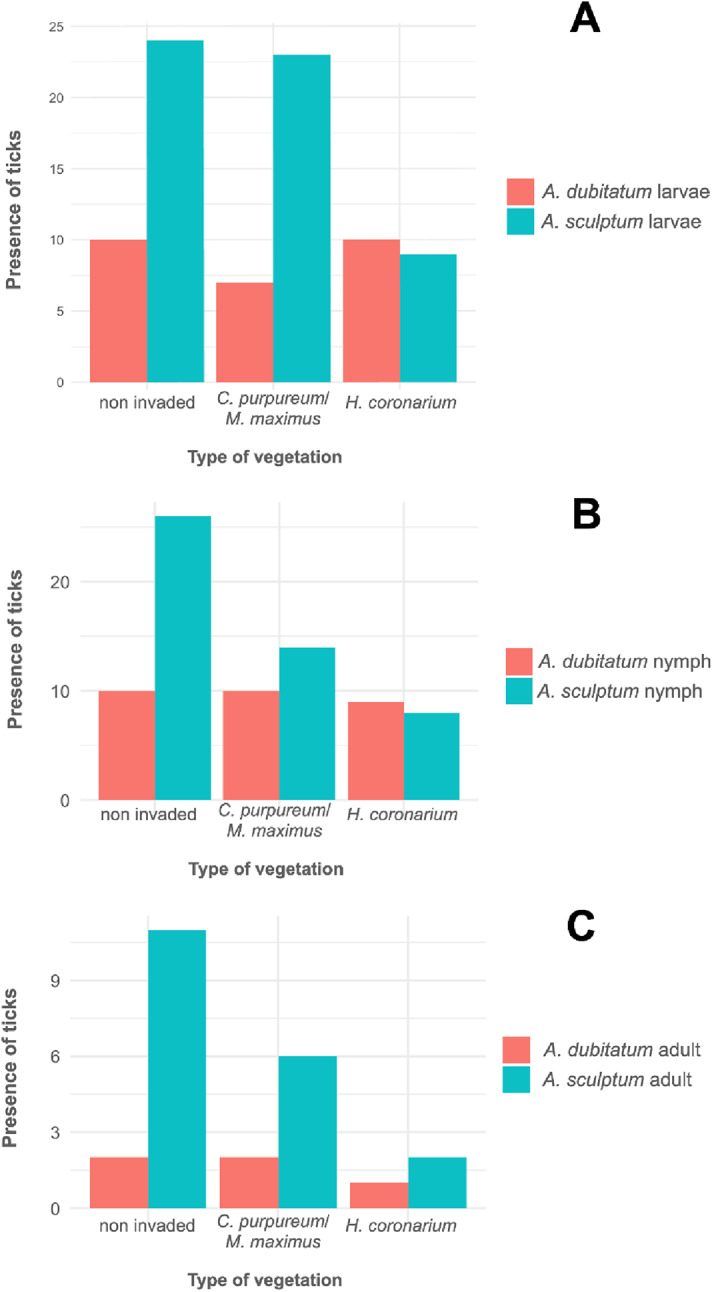
Representation of the number of sampled plots (25 m^2^ each) with the presence of larvae (**A**), nymphs (**B**) and adults (**C**) of *Amblyomma sculptum* and *Amblyomma dubitatum* in the three types of vegetation (H.cor: *Hedychium coronarium*; C.p/M.m: *Cenchrus purpureus* and *Megathyrsus maximus*; N.i.s.: non-invaded sites) sampled in the present study

**Fig. 3:**
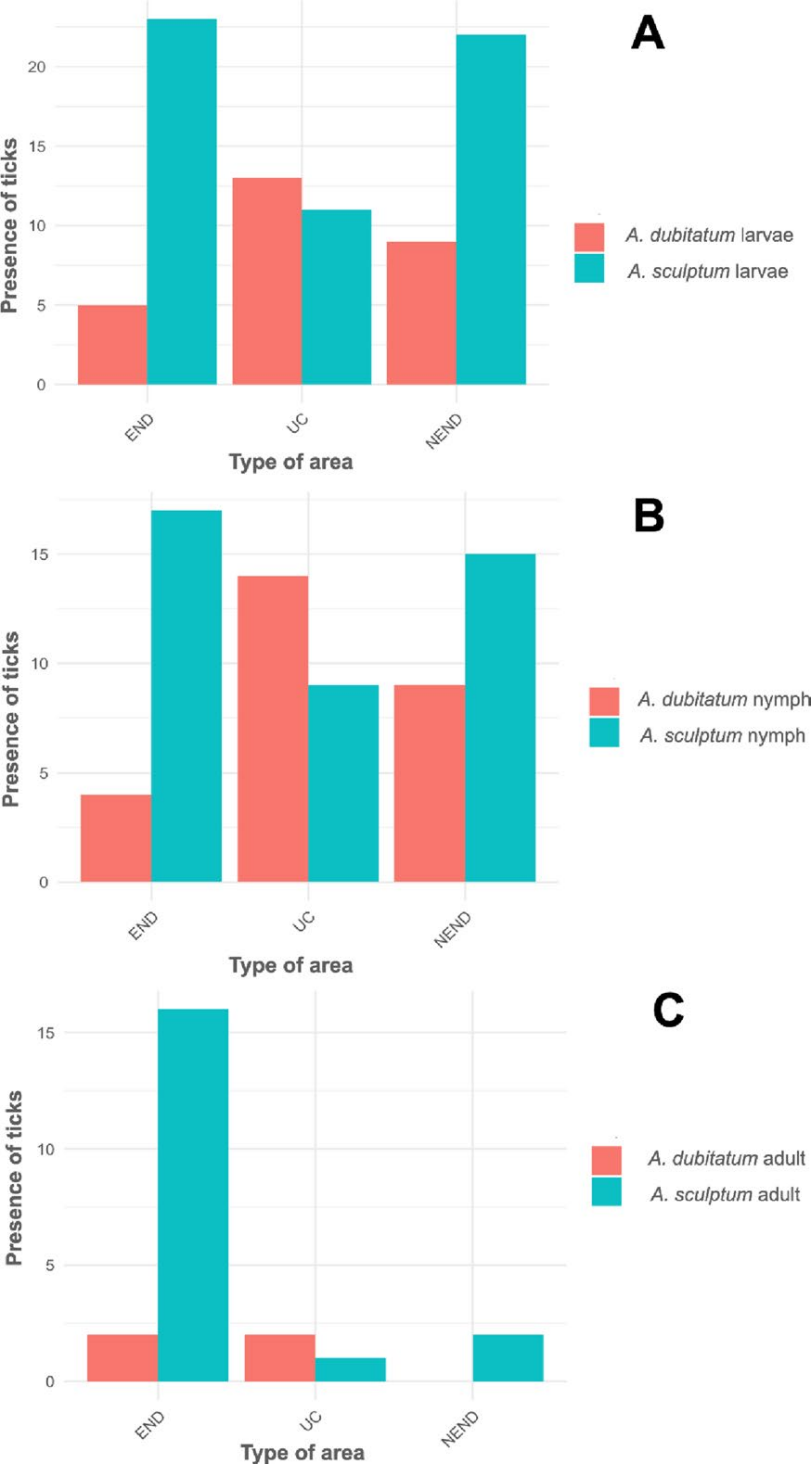
Representation of the number of sampled plots (25 m^2^ each) with the presence of larvae (**A**), nymphs (**B**) and adults (**C**) of *Amblyomma sculptum* and *Amblyomma dubitatum* in the three types of areas (END, NEND and UC) sampled in the present study. END and NEND consisted of human-modified landscape areas endemic or nonendemic, respectively, for Brazilian spotted fever; UC consisted of natural reserve areas within conservation units of the Atlantic Rainforest biome

Considering all three tick active stages together (larvae, nymphs and adults), the species *A. sculptum* was collected in all END and NEND areas, and in six UC areas, whereas *A. dubitatum* was collected in seven END areas, seven NEND areas, and in all UC areas; overall, the two tick species were collected together in 20 out of the 24 sampled areas (Table 2; Figs. 2 and 3; Supplementary Tables S1 to S3).

**Table 2:** Total number of larval clusters and individual nymphs and adults of *Amblyomma sculptum* and *Amblyomma dubitatum* per vegetation types (non-invaded sites, *Hedychium coronarium*, and *Cenchrus purpureus*/*Megathyrsus maximus*) and type of sampled area (END, NEND, and UC)

Vegetation type	Area type	Number of collected ticks					
		Larval clusters		Nymphs		Adults	
		*A. sculptum*	*A. dubitatum*	*A. sculptum*	*A. dubitatum*	*A. sculptum*	*A. dubitatum*
Non-invaded sites	END	73	0	48	0	28	1
	NEND	27	8	100	6	3	0
	UC	8	7	13	9	5	1
	Total	108	15	161	15	36	2
*H. coronarium*	END	6	14	0	0	7	0
	NEND	29	1	17	12	0	0
	UC	0	10	6	10	0	1
	Total	35	25	23	22	7	1
*C. purpureus/M. maximus*	END	41	6	241	7	21	1
	NEND	22	11	35	4	1	0
	UC	10	8	16	10	0	0
	Total	73	25	292	21	22	1

Through the analysis of tick presence as the response for type of predominating vegetation (non-invaded sites, *H. coronarium*, and *C. purpureus*/*M. maximus*), and type of area (END, NEND and UC), the only statistically significant association was a negative relationship between the presence of *A. sculptum* larvae and *H. coronarium*, when compared to *A. dubitatum* larvae (*p* = 0.0023); the latter tick species with presence statistically similar among all three vegetation types. Although the presence of *A. sculptum* nymphs and adults appeared higher in non-invaded sites than in *H. coronarium* and *C. purpureus*/*M. maximus*, there was no significant difference, as was also the case for *A. dubitatum*. The only vegetation type in which the presence of *A. dubitatum* was higher than *A. sculptum* was *H. coronarium* for larvae and nymphs. Although the number of sites with *A. sculptum* and *A. dubitatum* did not differ significantly in relation to the type of area (END, NEND and UC), the presence of all three stages of *A. sculptum* was visually much higher than *A. dubitatum* in both human-modified landscape areas (END and NEND), contrasting to UC areas where the presence of *A. dubitatum* surpassed *A. sculptum* for both larvae, nymphs and adults.

**Fig. 4:**
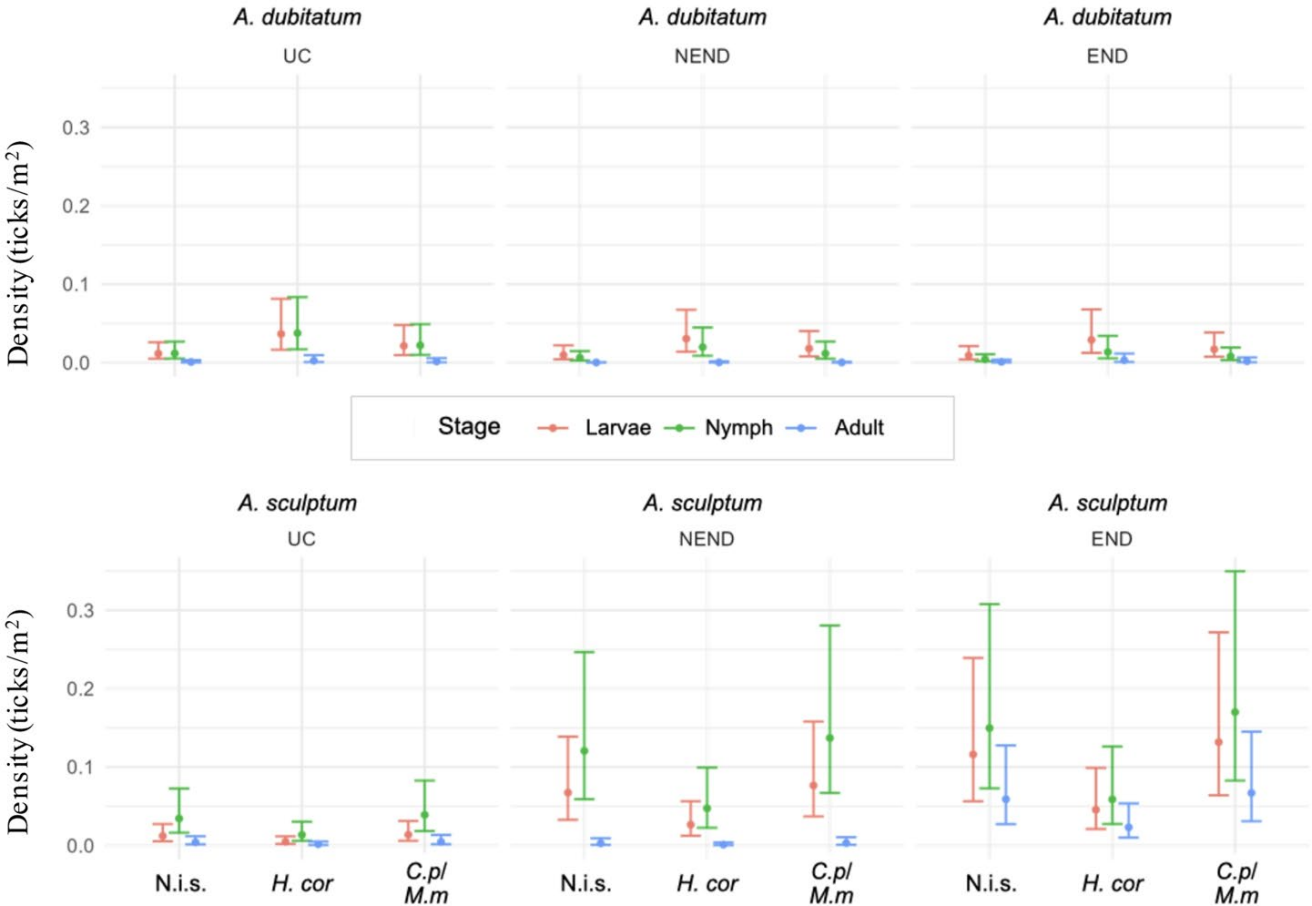
Estimated density and confidence intervals of larvae, nymphs and adults of *Amblyomma dubitatum* and *Amblyomma sculptum* in the three vegetation types (N.i.s.: non-invaded sites; *H.cor*: *Hedychium coronarium*; *C.p/M.m*: *Cenchrus purpureus* and *Megathyrsus maximus*) within the three types of areas (UC, NEND and END) sampled in the present study. In each vegetation type of each area, the total number of larval clusters, individual nymphs and adults were divided by the total area covered by flannel dragging to estimate the number of ticks per square meter (ticks/m^2^, here represented as un/m^2^)

**Fig. 5:**
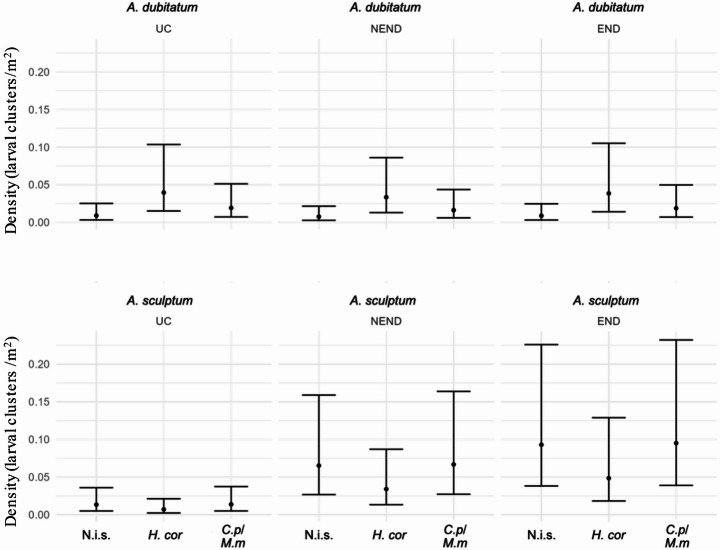
Estimated density and confidence intervals of larvae of *Amblyomma dubitatum* and *Amblyomma sculptum* in the three vegetation types (N.i.s.: non-invaded sites; *H.cor*: *Hedychium coronarium*; *C.p/M.m*: *Cenchrus purpureus* and *Megathyrsus maximus*) within the three types of areas (UC, NEND and END) sampled in the present study. In each vegetation type of each area, the total number of larval clusters were divided by the total area covered by flannel dragging to estimate the number of ticks per square meter (larval cluster/m^2^, here represented as un/m^2^)

**Fig. 6:**
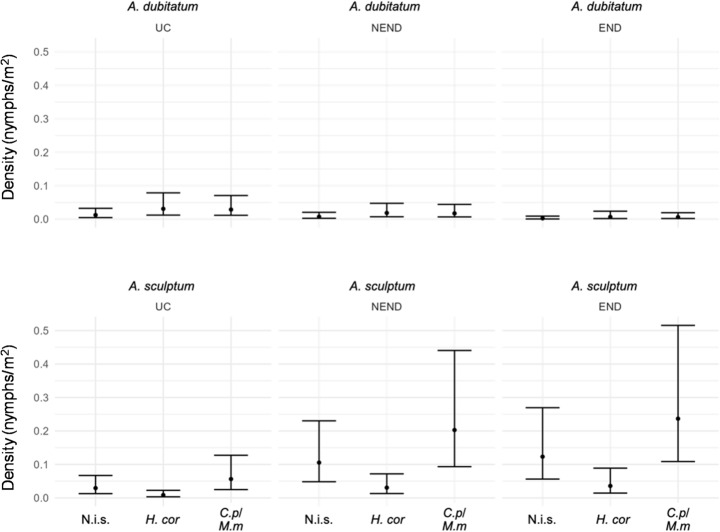
Estimated density and confidence intervals of nymphs of *Amblyomma dubitatum* and *Amblyomma sculptum* in the three vegetation types (N.i.s.: non-invaded sites; *H.cor*: *Hedychium coronarium*; *C.p/M.m*: *Cenchrus purpureus* and *Megathyrsus maximus*) within the three types of areas (UC, NEND and END) sampled in the present study. In each vegetation type of each area, the total number of collected nymphs were divided by the total area covered by flannel dragging to estimate the number of ticks per square meter (nymphs/m^2^)

The density analysis (ticks/m²) was performed based on the number of ticks collected and the size of each transect sampled (Supplementary Table S4). In general, mean densities (ticks/m²) of larvae, nymphs and adults were low in all combinations of tick species, vegetation types, and area types evaluated (Supplementary Data S1). The species *A. dubitatum* showed apparently less variation in density between different types of vegetation and area (Fig. 4). For *A. sculptum*, there was greater instability in distribution, since it presented very low densities in areas classified as UC, while higher values were observed in END areas (Fig. 4). In fact, the densities of *A. sculptum* larvae and nymphs were higher in areas classified as END and NEND than in the reference category (UC); they were also higher in END than in NEND areas (Figs. 5 and 6). Statistically, the densities of *A. sculptum* larvae were 7.12 times higher (IC 95%: 1.52–33.21; *p* = 0.01) and 5.76 times higher (IC 95%: 1.25–26.49; *p* = 0.02) in the END and NEND areas, respectively, than in the UC areas. Similarly, the densities of *A. sculptum* nymphs were 19.10 times higher (IC 95%: 4.11–88.69; *p* = 0.0001) and 5.96 times higher (IC 95%: 1.55–22.95; *p* = 0.009) in the END and NEND areas, respectively, than in the UC areas. Regarding adult ticks, only the END areas had density of *A. sculptum* adults significantly higher, which was 10.20 times higher (IC 95%: 1.14–91.04; *p* = 0.03) in the END than in the UC areas. The density of *A. dubitatum* larvae and nymphs did not appear to vary according to the type of area (UC, NEND, END), except for the nymphs of *A. dubitatum*, which had a 79% reduction of the density (IRR = 0.21; IC 95%: 0.06–0.74; *p* = 0.01) in END areas in relation to UC areas.

No significant difference in densities of adult ticks was observed when changing the type of vegetation. However, both larvae and nymphs of *A. sculptum* had lower densities in areas with *H. coronarium* vegetation, in which the density of *A. dubitatum* was higher, when compared to the reference category (non-invaded sites) (Figs. 5 and 6). Statistically, *A. sculptum* larvae had an 88% reduction of the density (IRR = 0.119; IC 95%: 0.02–0.61; *p* = 0.006) in *H. coronarium* in relation to non-invaded sites, whereas *A. sculptum* nymphs had an 89% reduction of the density (IRR = 0.110; IC 95%: 0.02–0.75; *p* = 0.004) in *H. coronarium* in relation to non-invaded sites. In contrast, the density of *A. dubitatum* larvae increased significantly in areas with *H. coronarium* vegetation, where it was 4.38 times higher (IC 95%: 1.45–13.20; *p* = 0.0087) than in the non-invaded sites.

The taxonomic identification of 12 larval clusters from UC areas was confirmed by molecular analysis. Partial sequences of the tick mitochondrial 16 S RNA gene were generated from 12 larval pools, of which eight were identified as *A. sculptum* and four as *A. dubitatum* (Supplementary Table S5). These *A. sculptum* pools were from the following areas (vegetation type): UC1 (one pool on *M. maximus*), UC2 (two pools on *M. maximus* and one pool on non-invaded site), UC3 (two pools on *M. maximus*), UC4 (one pool on *C. purpureus*) and UC8 (one pool on *M. maximus*). The four *A. dubitatum* pools were from UC5 (two pools on *H. coronarium*) and UC6 (one pool on *H. coronarium* and one pool on non-invaded site).

Besides *A. sculptum* and *A. dubitatum*, the only other tick species identified in the present study were *Amblyomma ovale* (one nymph from END6) and *Amblyomma coelebs* (10 nymphs from UC1, UC2 and UC3).

## Discussion

Based on tick collections from riparian forests inhabited by capybaras, the species *A. sculptum* and *A. dubitatum* were found in sympatry in 14 out of 16 human-modified landscape areas, and in six out of eight natural reserve areas sampled. These results corroborate previous studies that showed a predominant co-occurrence of these two tick species in human-modified landscapes inhabited by capybaras (Souza et al. 2006; Queirogas et al. 2012; Brites-Neto et al. 2013; Luz et al. 2019). Our results showed that the density of *A. sculptum* was greater in human-modified landscapes, while the density of *A. dubitatum* tended to be similar among human-modified landscapes and natural reserve areas. In addition, the presence/absence analysis of *A. dubitatum* also did not differ among human-modified landscapes and natural reserve areas. These results suggest that *A. dubitatum* is generally less affected by human activity than *A. sculptum* is. During an eight-year period in a residential park in the state of São Paulo, Passos Nunes et al. (2019) observed that a 3-fold increase of the capybara population was accompanied by a 40-fold increase of the *A. sculptum* population; however, *A. dubitatum* remained approximately stable during the same period. Because there was no marked change in the landscape of the capybara-inhabited areas in the residential park, the authors concluded that the tremendous increase of the *A. sculptum* population was directly linked to the increase of capybara density during the eight-year period (Passos Nunes et al. (2019). These results were corroborated by two complementary studies in the state of São Paulo that reported significantly higher *A. sculptum* densities in human-modified landscapes with significantly higher capybara abundance and densities (Luz et al. 2019; Lopes 2021). Based on these previous studies and despite not quantifying capybara populations in the 24 study areas, the higher density of *A. sculptum* in human-modified landscapes of the present study was likely due to capybara abundance rather than landscape modification alone.

Regarding the three vegetation types analyzed in this study, it is noteworthy that *H. coronarium* was clearly less favorable for *A. sculptum* than the other vegetation types. In addition, the only vegetation type in which the presence of *A. dubitatum* was higher than *A. sculptum* was *H. coronarium* for larvae and nymphs [this difference was not observed for adults possibly because it has been shown that flannel drag is less efficient for collecting adults of *A. dubitatum* (Szabó et al. 2024)]. These results could be related to the fact that *H. coronarium* is associated with more humid soils, usually seasonally flooded (Vergne et al. 2023). Field studies in capybara-living areas have reported that *A. dubitatum* is usually associated with more humid soils than *A. sculptum* (Szabó et al. 2007; Queirogas et al. 2012; Pajuaba Neto et al. 2018). These observations have been corroborated by laboratory assays that showed greater tolerance of all developing stages of *A. dubitatum* to water immersion than *A. sculptum*; *A. sculptum*, by contrast, is more tolerant of lower relative humidities than *A. dubitatum* (Luz et al. 2020). As the establishment of *H. coronarium* promotes soil moisture due to lower evapotranspiration (Vergne et al. 2023), the presence of this invasive macrophyte may be unfavorable to *A. sculptum*.

It is intriguing that the presence and density of *A. dubitatum* was not altered by the type of area (UC, NEND, and END) in nearly all analyses. Based on the study by Passos-Nunes et al. (2019), it is possible that the increase in capybara density may not influence *A. dubitatum* density because increasing capybara density does not necessarily increase the area of wet soil occupied (seasonally flooded soils favorable to *A. dubitatum*). On the other hand, higher capybara densities lead to the occupation of larger dry areas with food sources for capybaras, such as grasses and various points in the forest further away from the flooded areas near the water’s edge. Thus, the association between the increase in *A. sculptum* populations and the increase in capybara populations (Luz et al. 2019; Passos Nunes et al. 2019; Lopes 2021) may be related to the increased use of areas favorable to ticks, such as invaded grasses (*M. maximus* or *C. purpureus*).

This study showed that among human-modified landscapes, there were higher *A. sculptum* densities in BSF-endemic areas (END) than in non-endemic areas (NEND). Although we did not quantify the capybara groups in the 24 areas of the present study, this result related to *A. sculptum* also suggests that there was a higher capybara density in the BSF-endemic areas than in non-endemic areas, as previously reported for seven human-modified landscape areas of the state of São Paulo (Luz et al. 2019; Lopes 2021). In fact, the same areas of the study of Luz et al. (2019) and Lopes (2021) were included in the 16 human-modified landscape areas that were sampled during the present study. These results support previous studies that indicated that in order to maintain *R. rickettsii* infection in a population of *A. sculptum* (endemic to BSF), both an overgrowth population of *A. sculptum* and a high reproduction rate of capybaras are necessary (Polo et al. 2017, 2018; Ramírez-Hernández et al. 2020).

In our study, each area was sampled only once. Although sampling was directed toward the peak period of immature stages (especially larvae), a single sampling may have reduced the sensitivity of tick detection, contributing to large numbers of transects yielding zero ticks (Tables S1-S3). Thus, the fact that *A. sculptum* was not collected in two of the eight UC areas, and *A. dubitatum* in two of the 16 human-modified landscape areas, does not necessarily mean that they did not exist in these areas; they might have been present in densities below the detection threshold for a single sampling day.

## Conclusions

In conclusion, this study across 24 capybara-inhabited landscapes revealed that the ticks *A. sculptum* and *A. dubitatum* commonly occurred together, but their abundance and distribution varied with human landscape modification and vegetation type. *Amblyomma sculptum* was denser in human-modified landscapes, especially in BSF-endemic areas. On the other hand, *A. dubitatum* remained more constant across both natural reserve and human-modified landscapes areas. Notably, invasive vegetation such as *H. coronarium* supported higher densities of *A. dubitatum* larvae and nymphs but suppressed *A. sculptum*. These patterns suggest that environmental changes, particularly plant invasions and capybara-driven habitat transformation, significantly influence the ecology of these medically important ticks.

## Supplementary Information

Below is the link to the electronic supplementary material.

Supplementary Material 1

Supplementary Material 2

Supplementary Material 3

## Data Availability

We declare all data is being provided within this manuscript.
