# Supplementary material for: Assessment of tick populations associated with capybaras in natural reserves and human-modified environments with or without invasive plants in the state of São Paulo, Brazil
Source: Exp Appl Acarol. 2026 Mar 14;96(3):34. doi: 10.1007/s10493-026-01127-w (PMC12988901; doi:10.1007/s10493-026-01127-w)
Supplement: Supplementary file 1 — Supplementary Material 1 [file 10493_2026_1127_MOESM1_ESM.pdf]

Supplementary Figure S1. Aerial views of the 24 areas visited in the state of São Paulo, where ticks were collected in this study. Eight areas (END1 to END8) consisted of anthropized areas endemic for Brazilian spotted fever; eight areas (NEND1 to NEND8) consisted of anthropized areas not endemic for Brazilian spotted fever; and eight areas (UC1 to UC8) consisted of natural landscapes within conservation units of the Atlantic Forest or Cerrado biomes. In each area, ticks were sampled in non-invaded sites (N.i.s.), which were sites dominated by native vegetation without exotic grasses and *Hedychium coronarium*. When present, sites dominated by *H. coronarium* (H. cor) and sites dominated by either *Cenchrus purpureus* or *Megathyrsus maximus* (C.p/M.m) grasses were also sampled. In each area, ticks were collected by dragging a flannel cloth along a 75 m trail, indicated by colored lines in each image. Satellite images obtained from Google LLC. (2025). Google Earth Pro - Version: 7.3.6.10201 (64 bits) [Mac OS X (15.7.0)]. Google. https://www.google.com/ earth/

END1

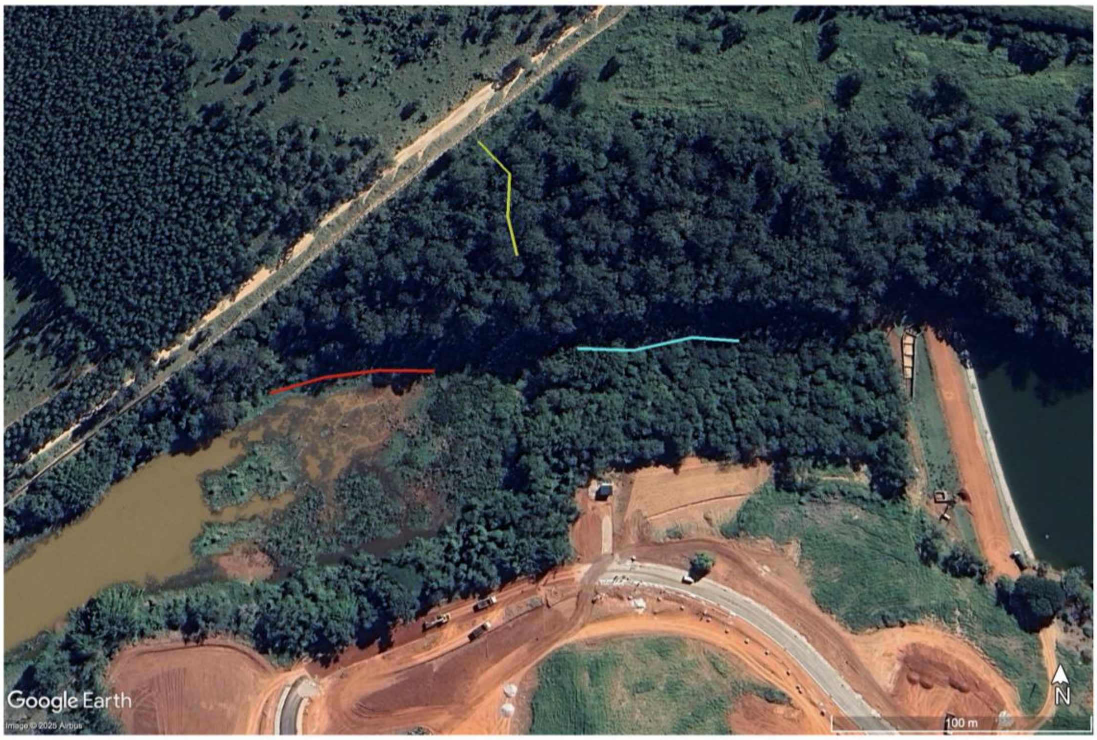

- *H. cor*
- *C.p/M.m*
- N.i.s.

END2

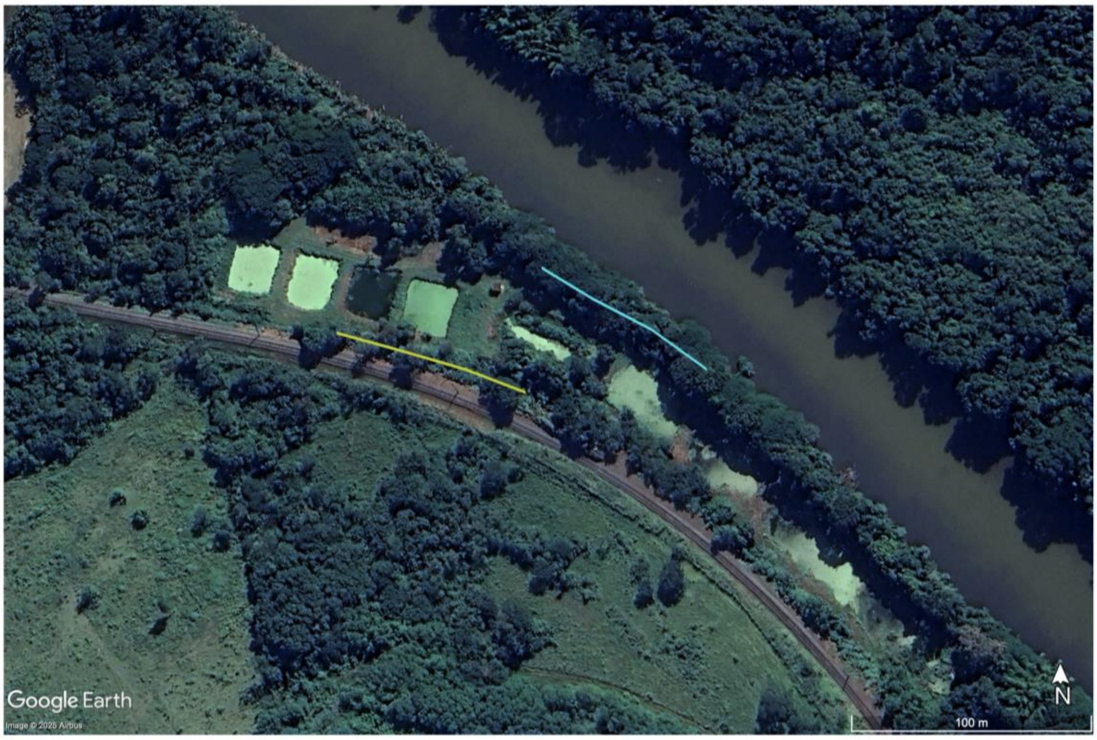

- *C.p/M.m*
- N.i.s.

END3

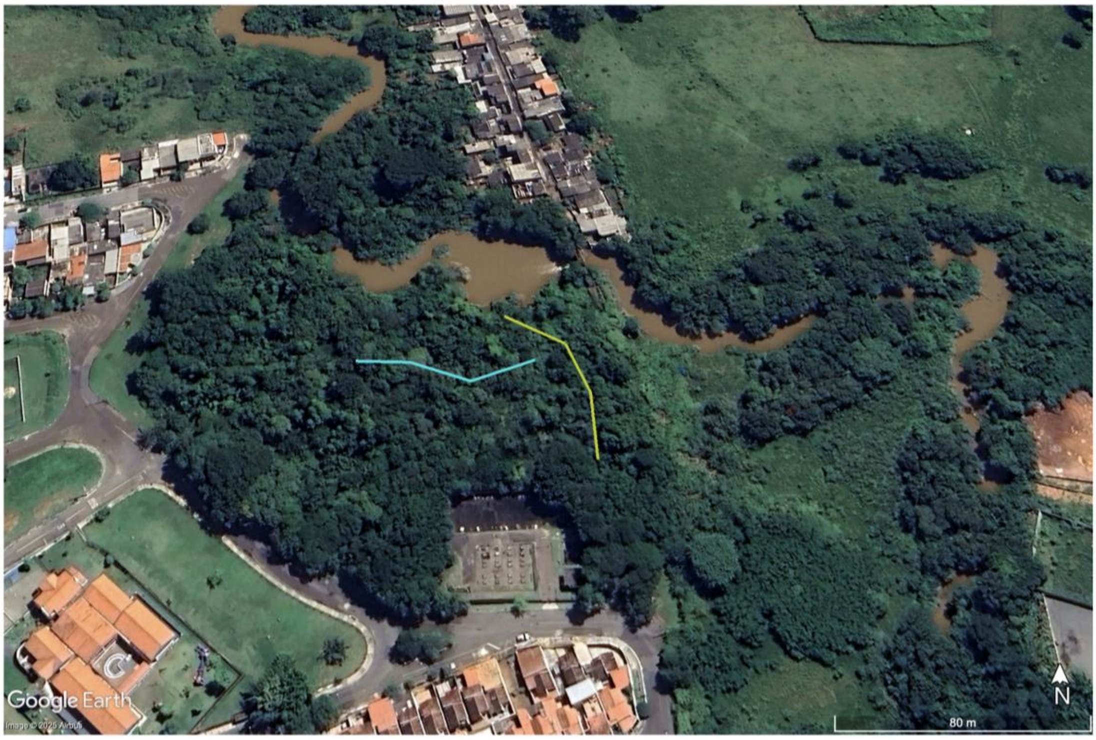

C.p/M.m
N.i.s.

END4

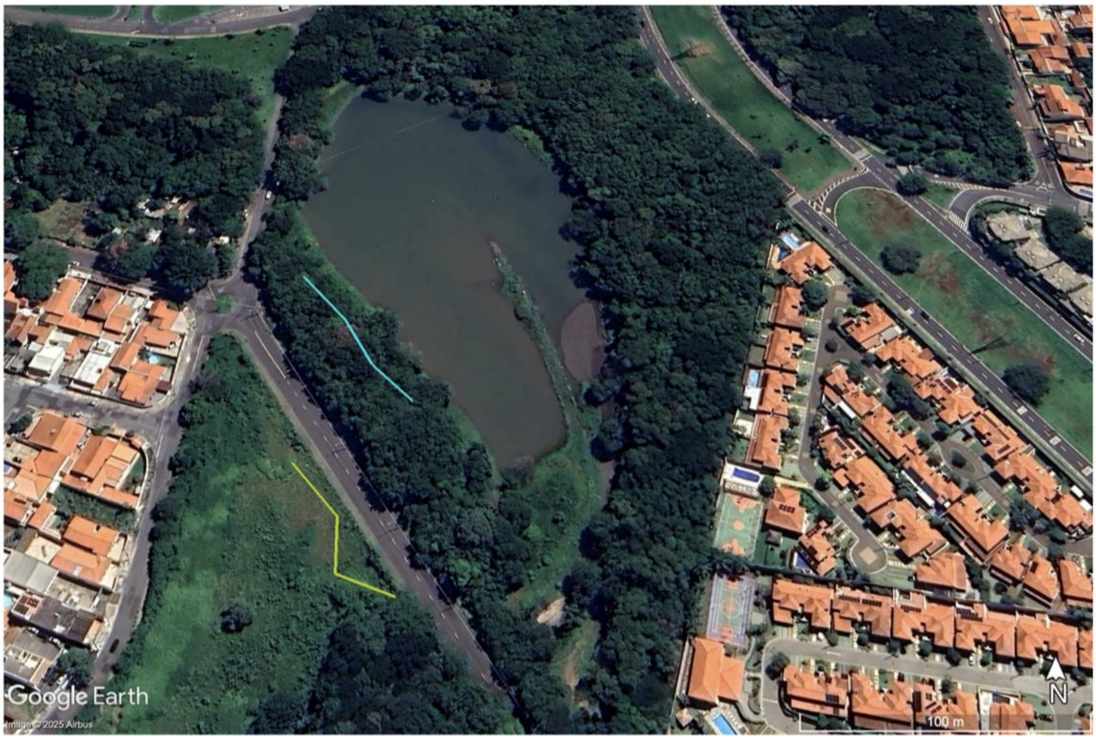

C.p/M.m
N.i.s.

END5

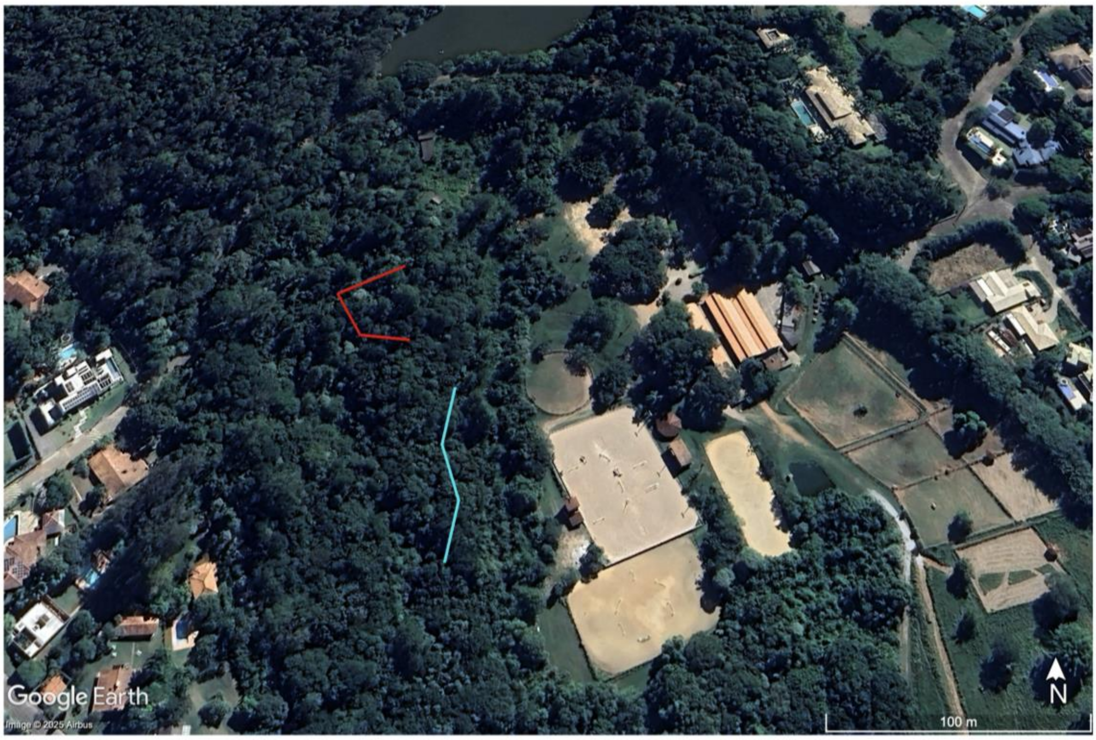

H. cor
N.i.s.

END6

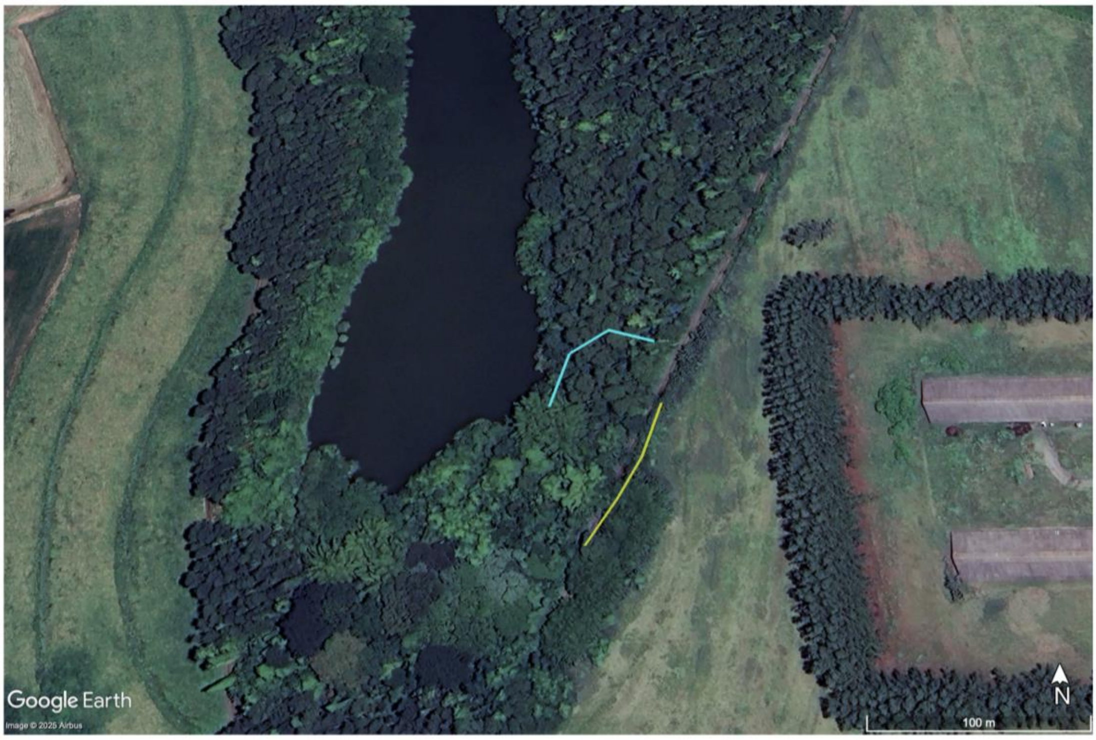

C.p/M.m
N.i.s.

END7

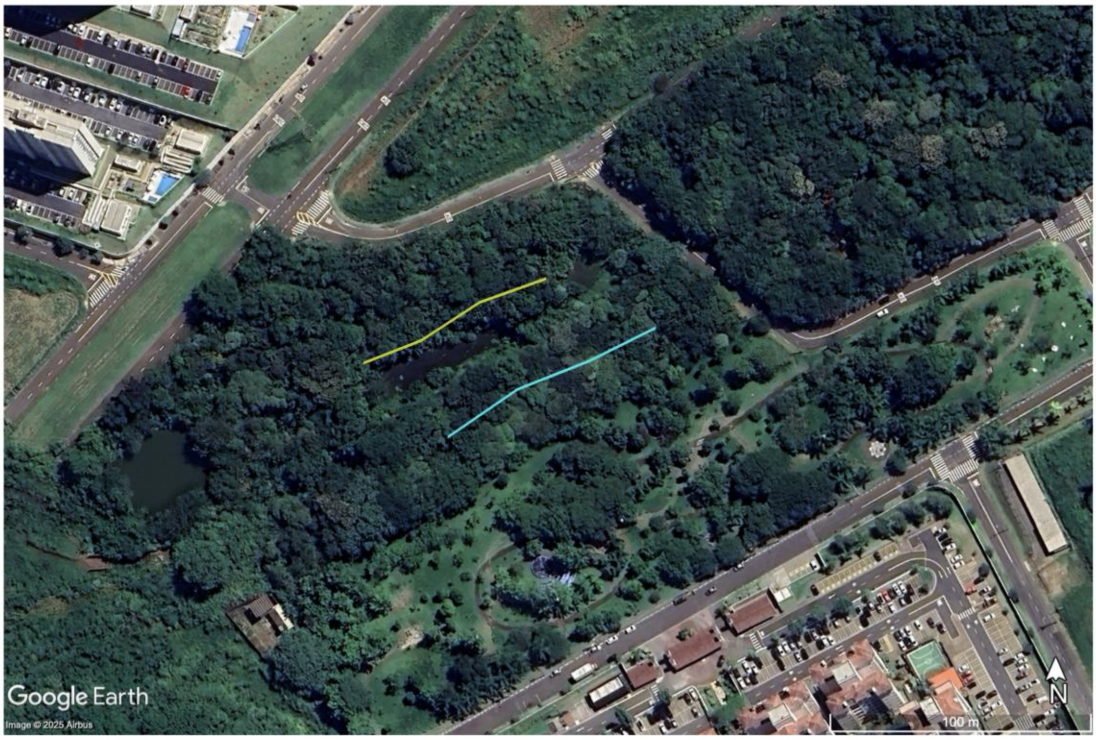

C.p/M.m
N.i.s.

END8

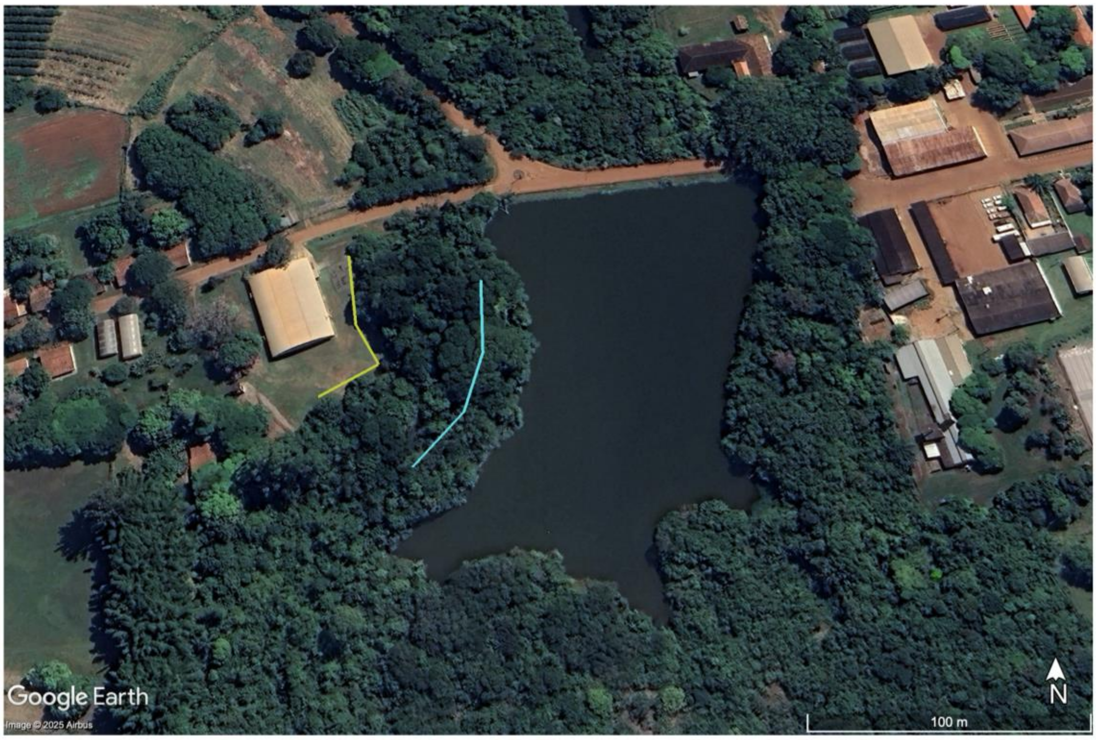

C.p/M.m
N.i.s.

NEND1

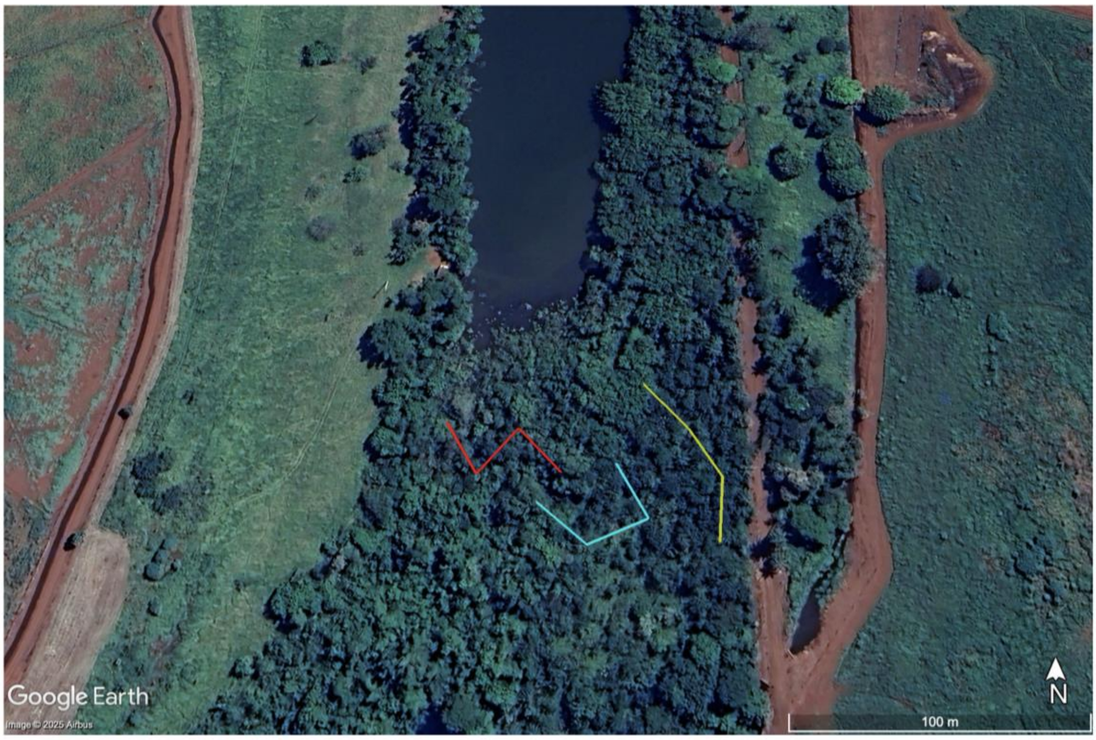

H. cor
C.p/M.m
N.i.s.

NEND2

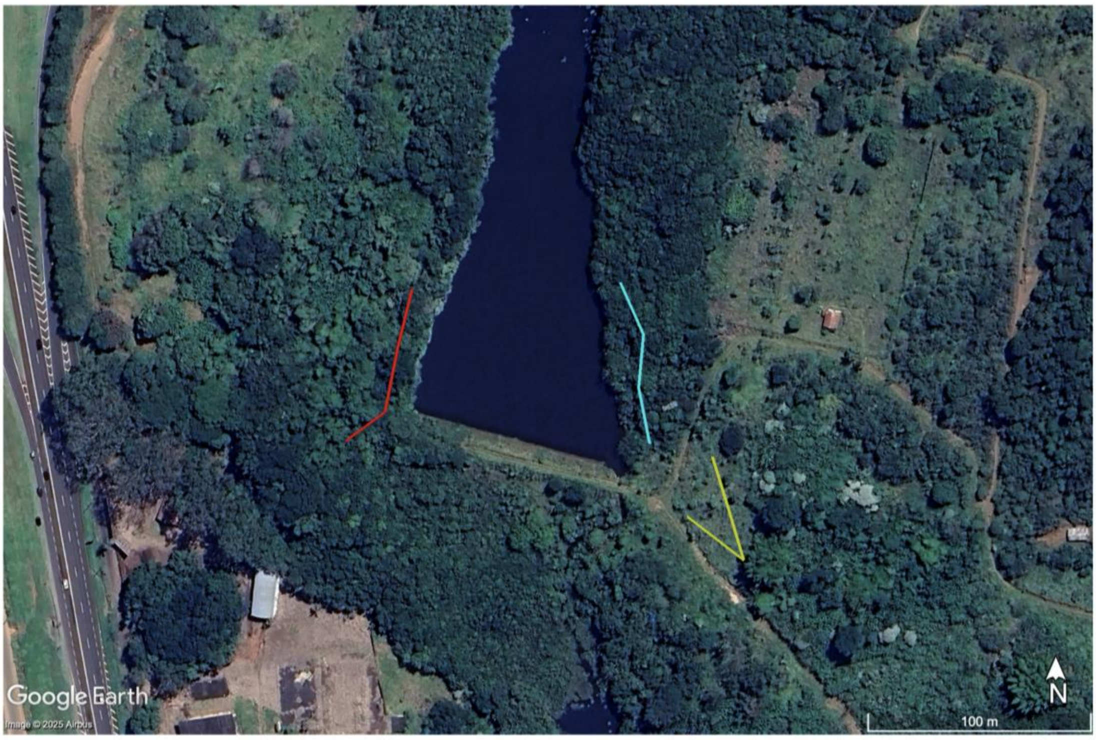

H. cor
C.p/M.m
N.i.s.

NEND3

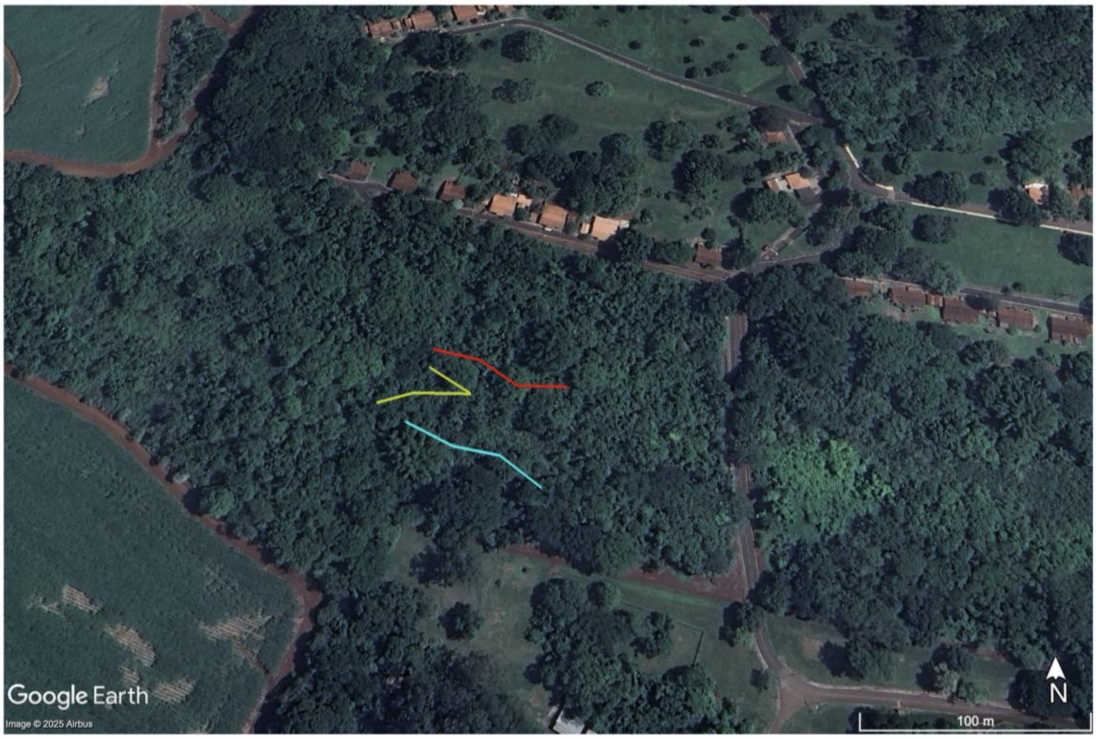

H. cor
C.p/M.m
N.i.s.

NEND4

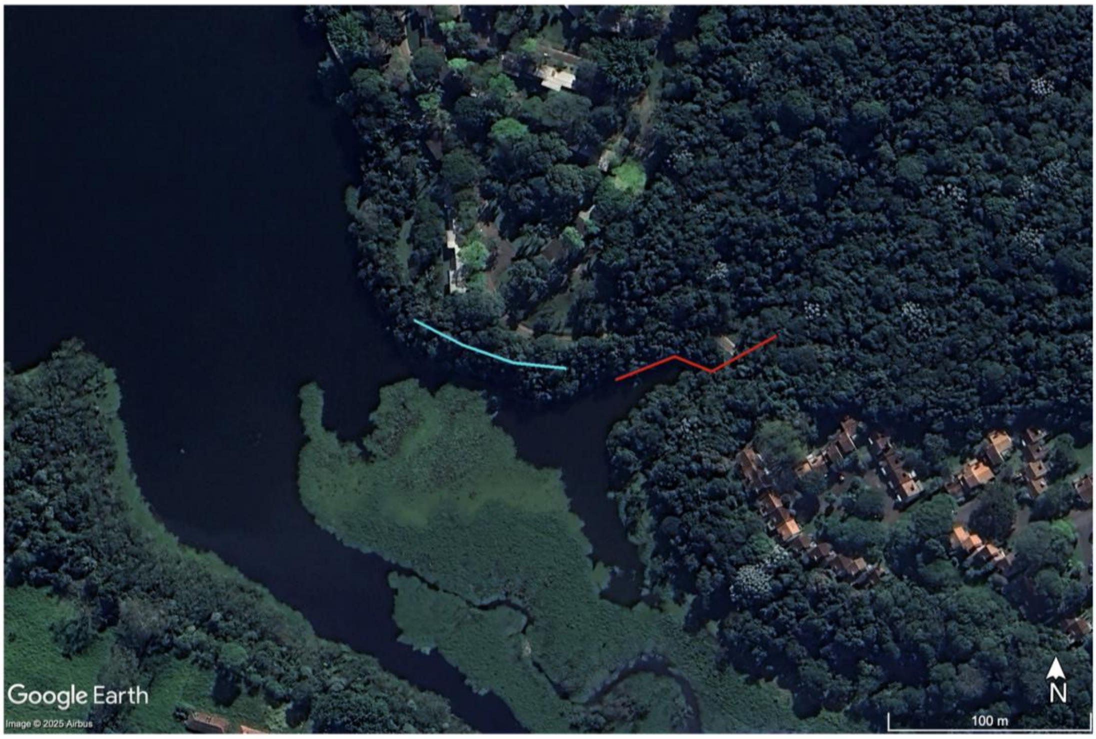

H. cor
N.i.s.

NEND5

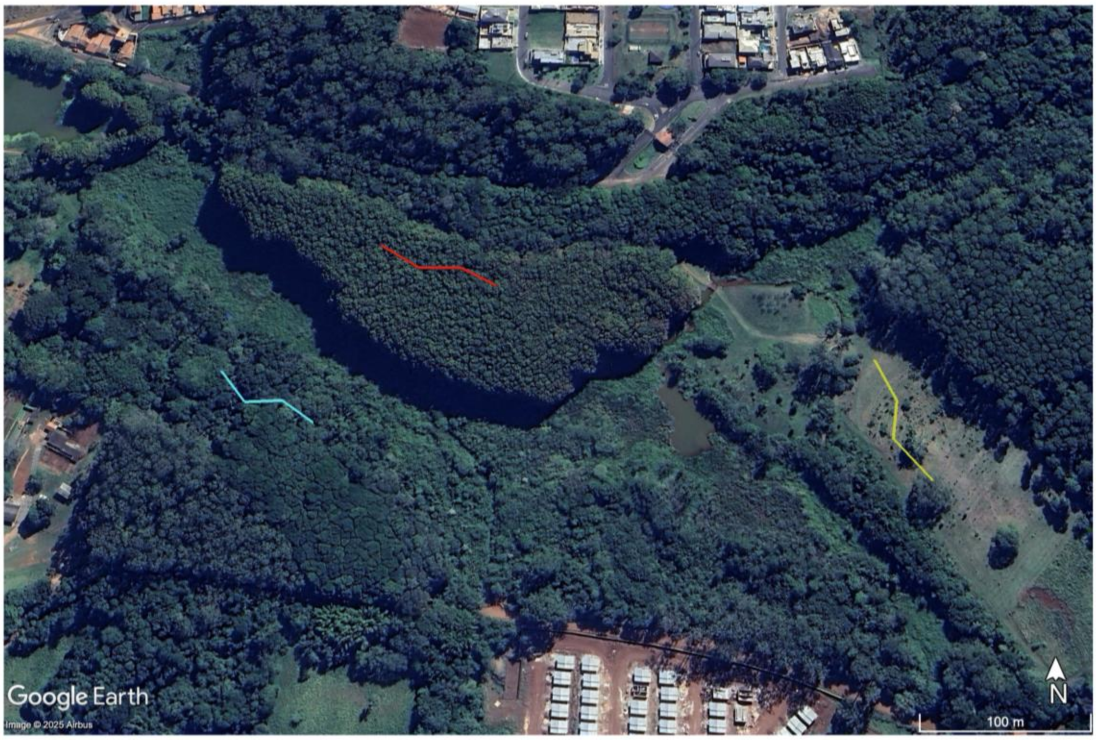

H. cor
C.p/M.m
N.i.s.

NEND6

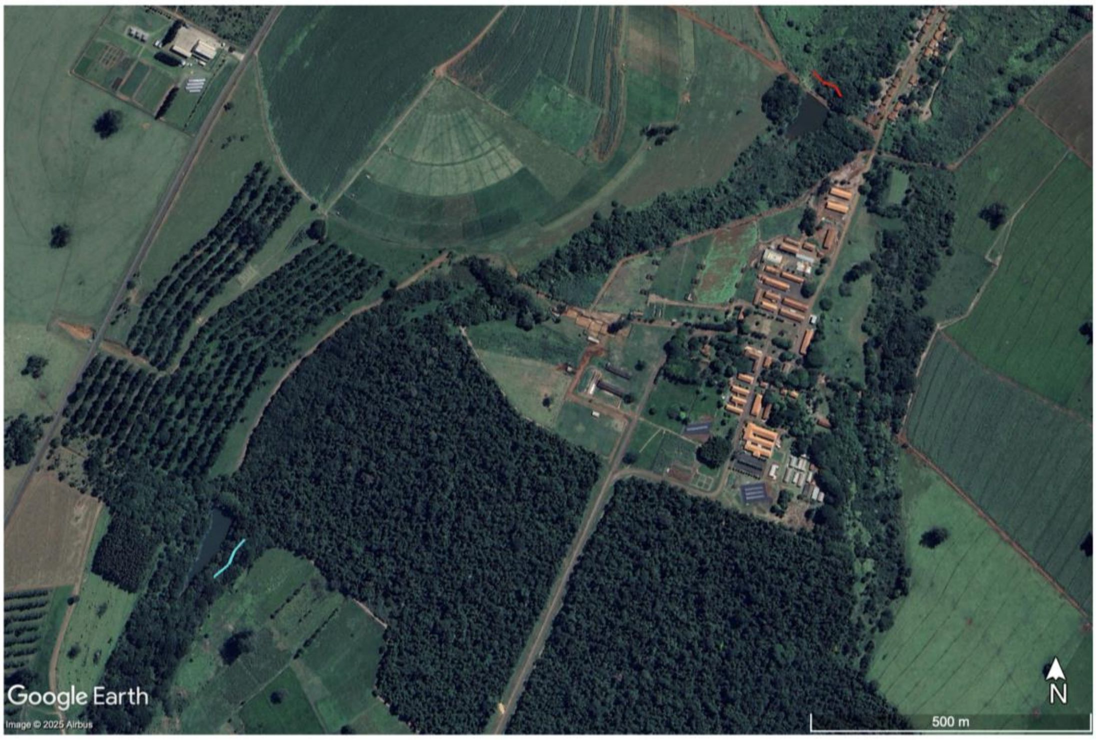

H. cor
N.i.s.

NEND7

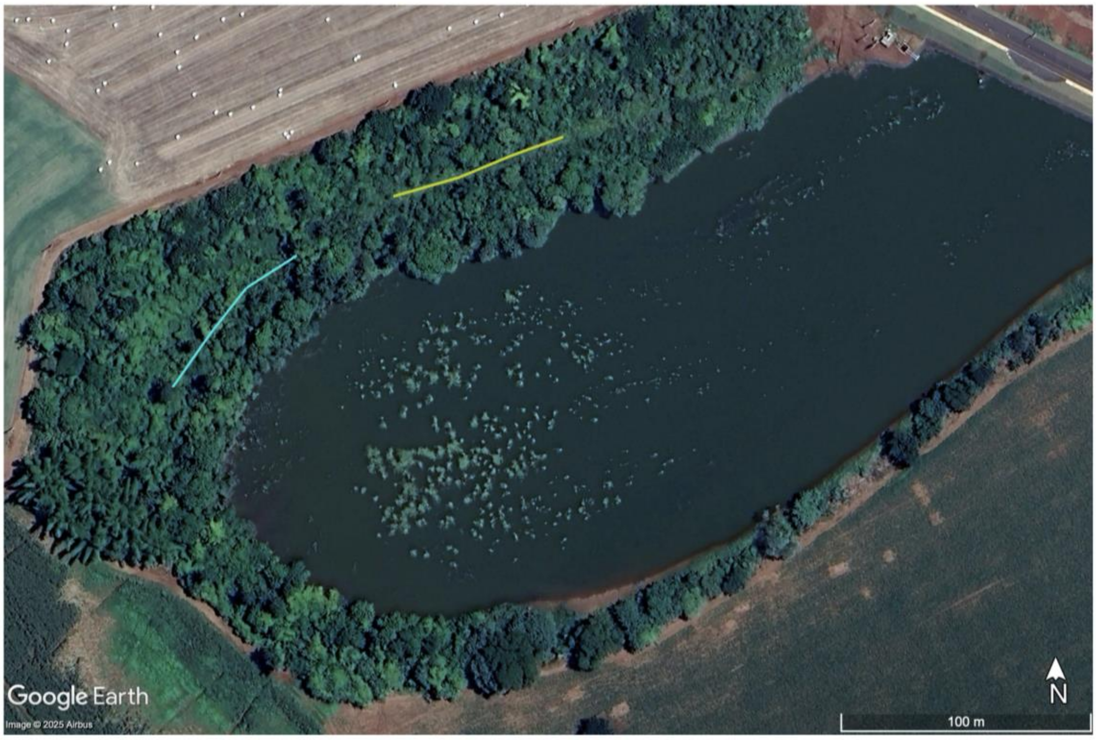

C.p/M.m
N.i.s.

NEND8

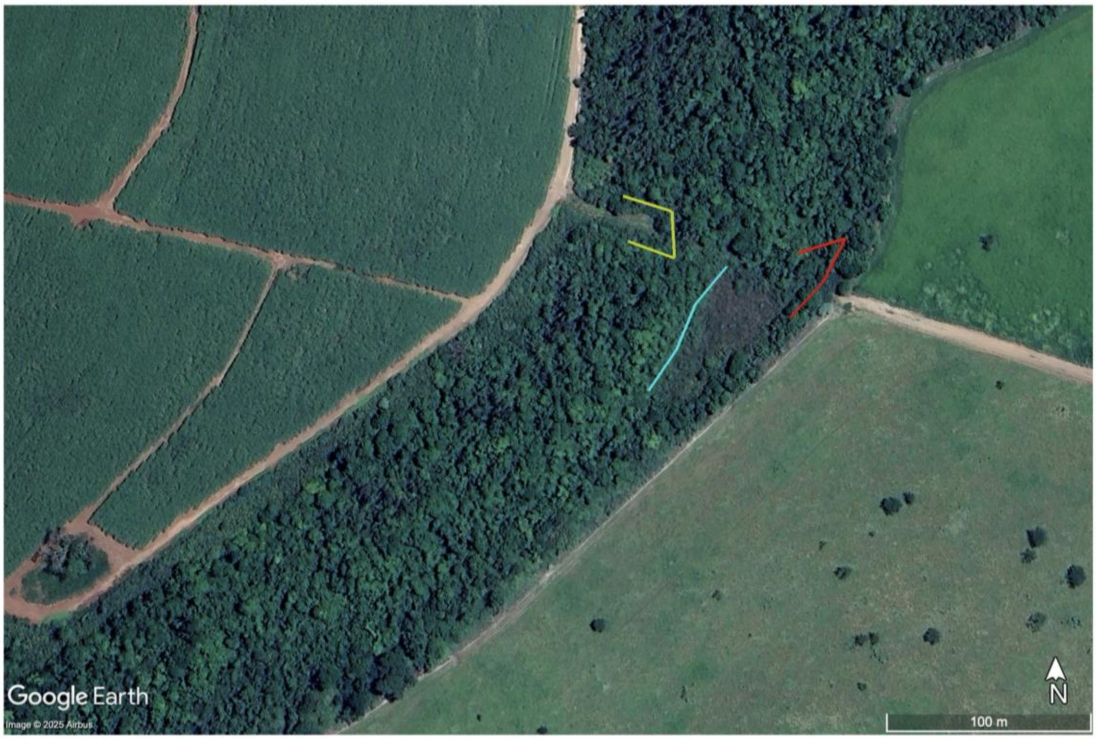

H. cor
C.p/M.m
N.i.s.

UC1

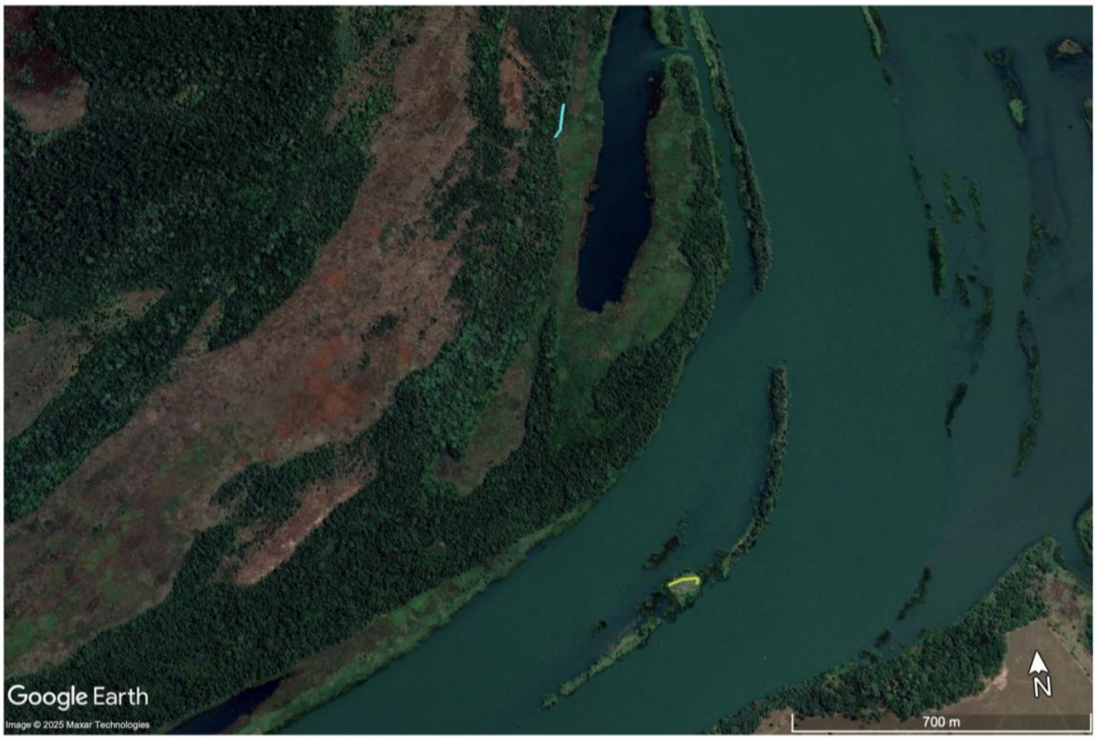

C.p/M.m
N.i.s.

UC2

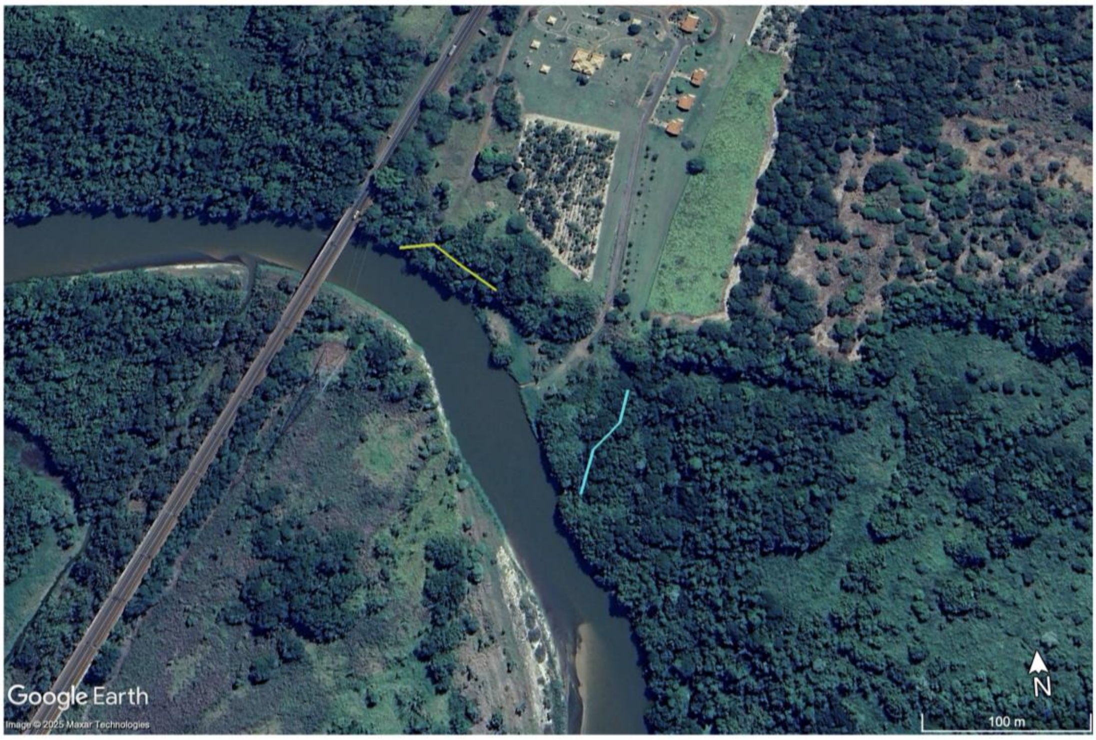

C.p/M.m
N.i.s.

UC3

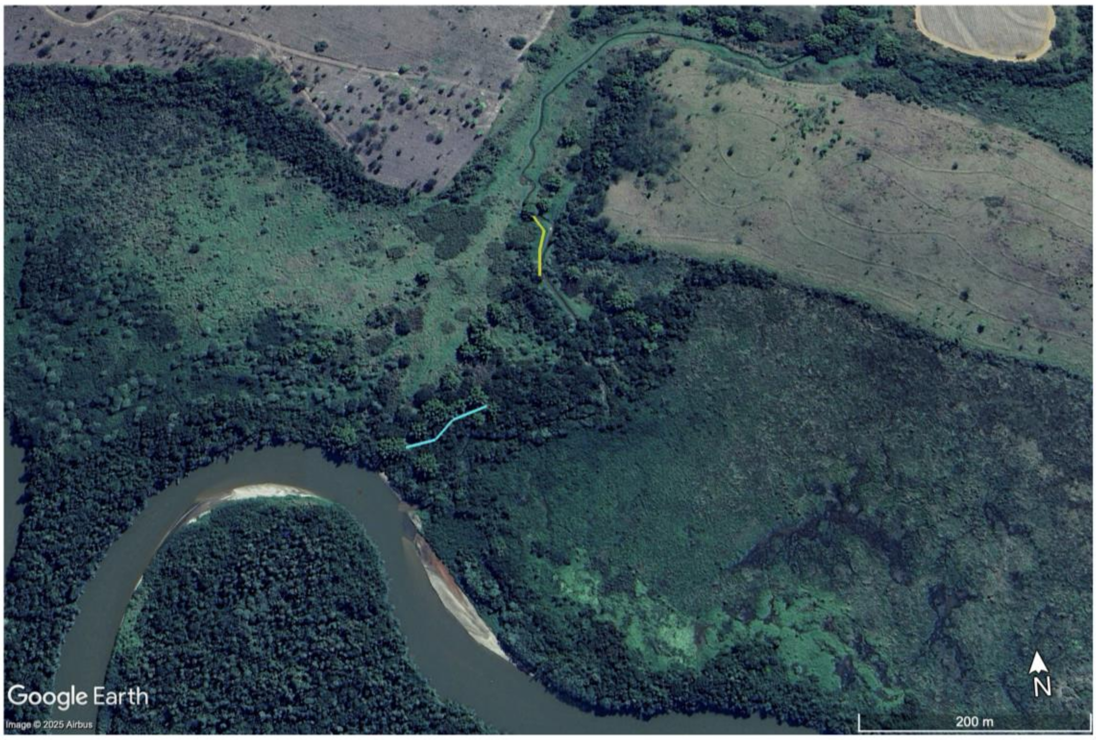

C.p/M.m
N.i.s.

UC4

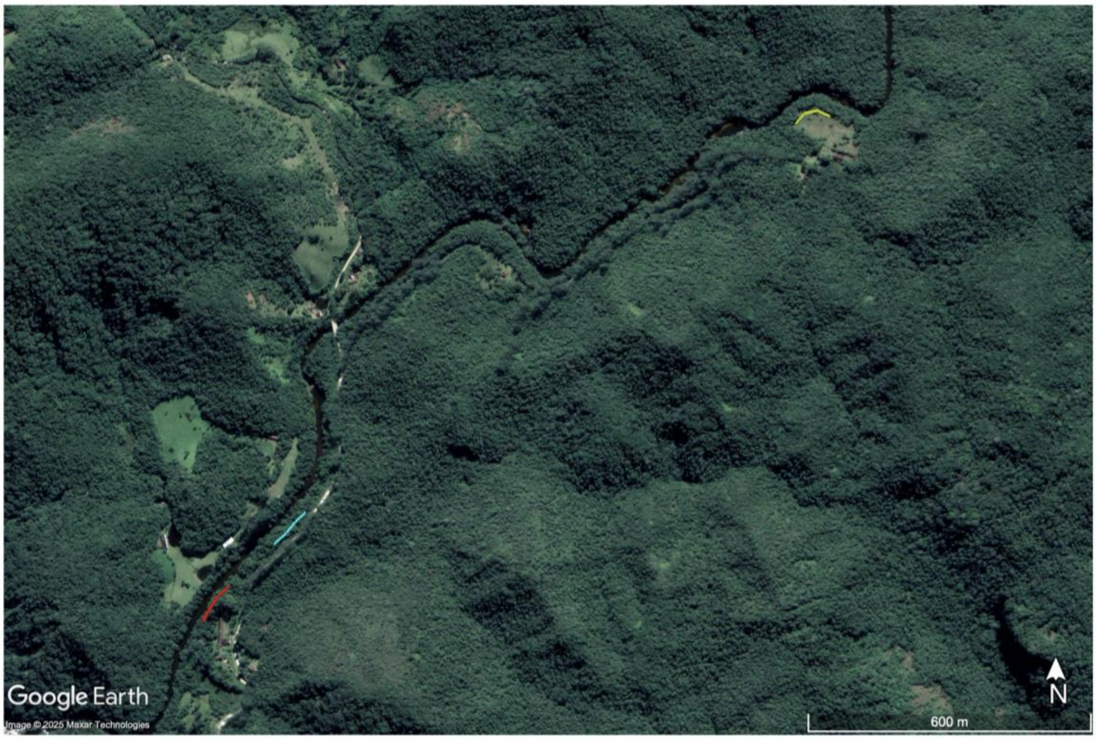

H. cor
C.p/M.m
N.i.s.

UC5

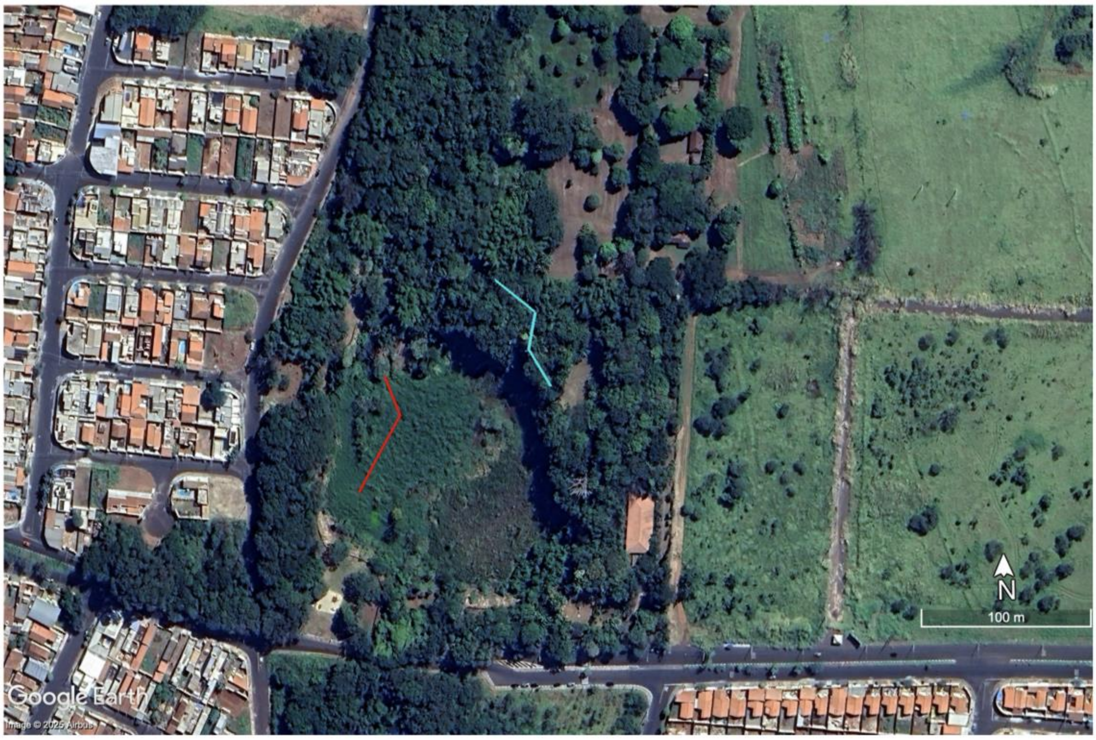

H. cor
N.i.s.

UC6

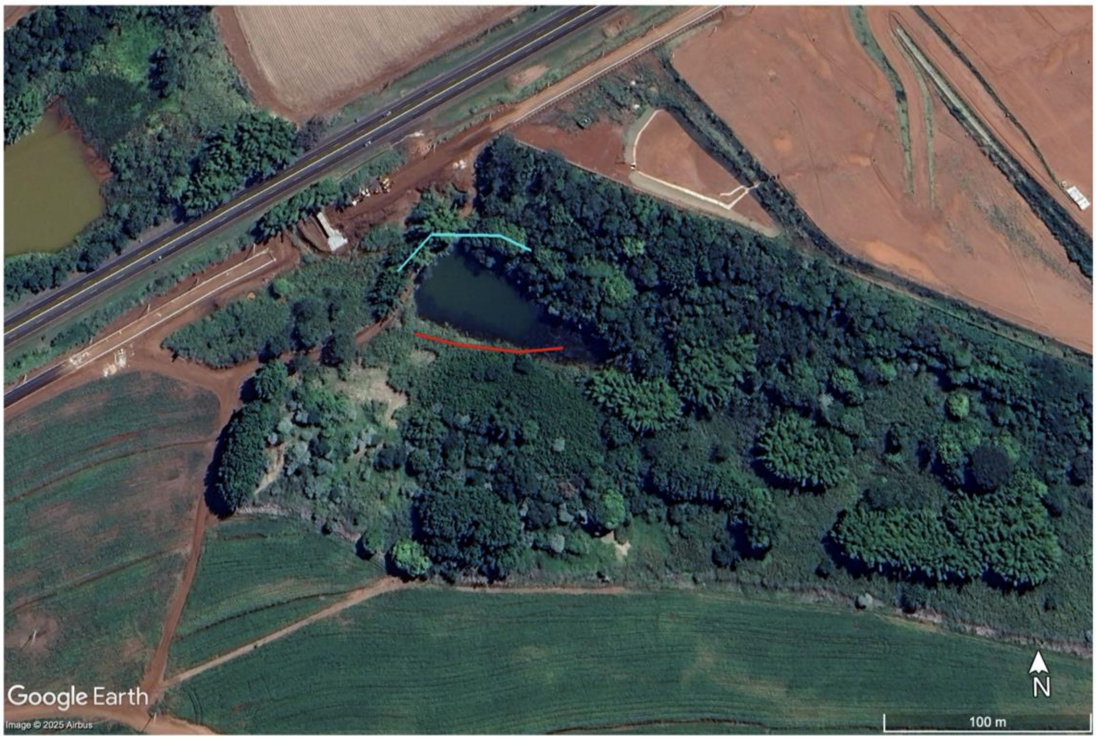

H. cor
N.i.s.

UC7

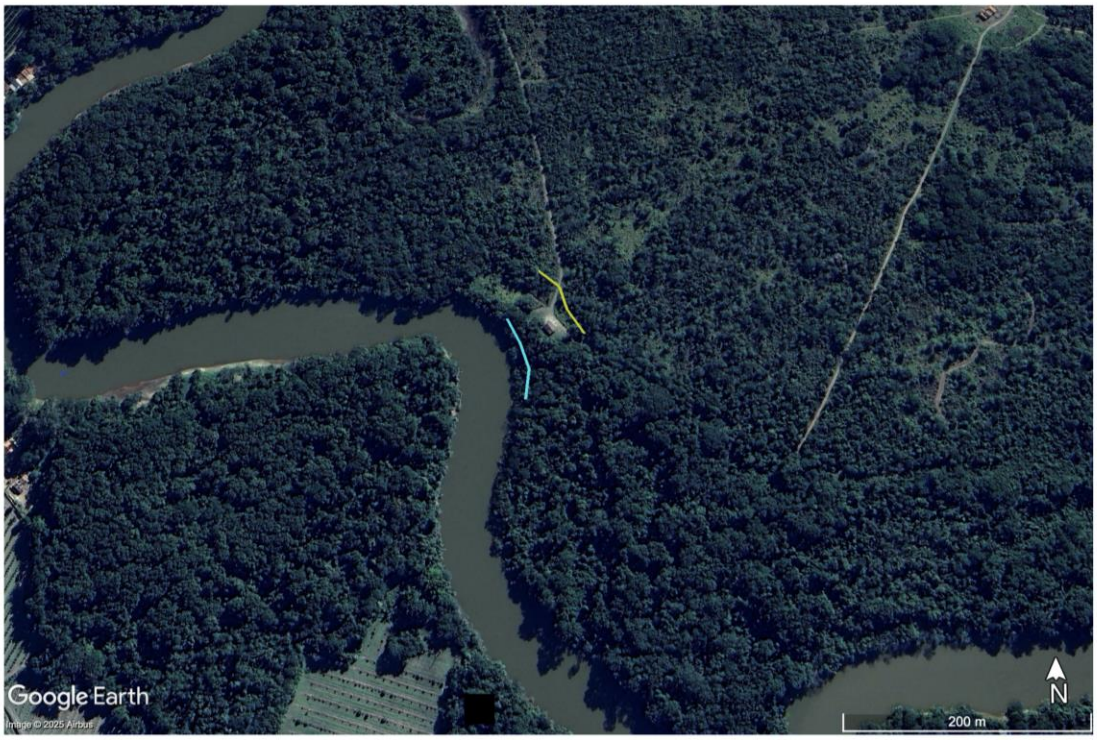

C.p/M.m
N.i.s.

UC8

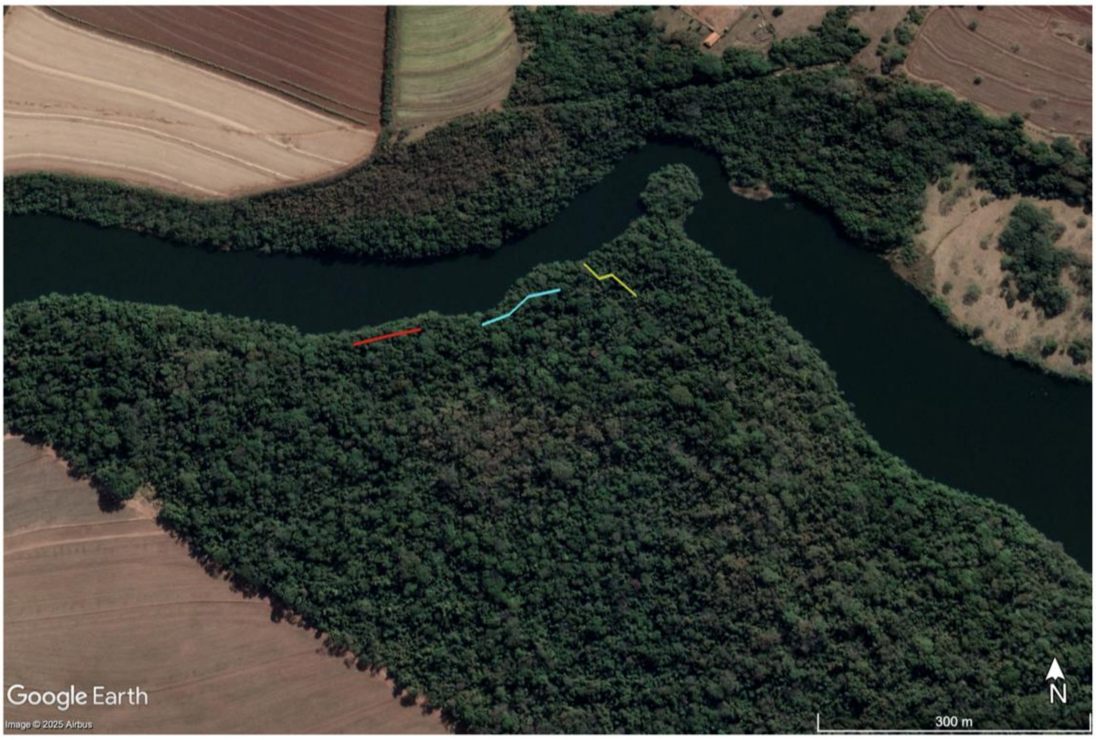

H. cor
C.p/M.m
N.i.s.
