# Supplementary material for: Assessment of tick populations associated with capybaras in natural reserves and human-modified environments with or without invasive plants in the state of São Paulo, Brazil
Source: Exp Appl Acarol. 2026 Mar 14;96(3):34. doi: 10.1007/s10493-026-01127-w (PMC12988901; doi:10.1007/s10493-026-01127-w)
Supplement: Supplementary file 2 — Supplementary Material 2 [file 10493_2026_1127_MOESM2_ESM.docx]

Table S1. Number of larvae (larval clusters) and individual nymphs and adults of *Amblyomma sculptum* and *Amblyomma dubitatum* per vegetation types (non-invaded sites, *Hedychium coronarium*, *Cenchrus purpureus* and *Megathyrsus maximus*) from eight human-modified landscape areas (END 1 to END8) endemic for Brazilian spotted fever in the state of São Paulo, Brazil.

| Areas | Vegetation type | Number of collected ticks | | | | | |
| --- | --- | --- | --- | --- | --- | --- | --- |
|  |  | *A.sculptum* larvae | *A.dubitatum* larvae | *A.sculptum* nymph | *A.dubitatum* nymph | *A.sculptum* adult | *A.dubitatum* adult |
| END1 | Non-invaded | 0 | 0 | 12 | 0 | 4 | 0 |
|  | *M. maximus* | 0 | 0 | 113 | 3 | 3 | 0 |
| END2 | Non-invaded | 1 | 0 | 0 | 0 | 4 | 0 |
|  | *C. purpureus* | 0 | 0 | 0 | 0 | 3 | 0 |
|  | *H. coronarium* | 2 | 4 | 0 | 0 | 6 | 0 |
| END3 | Non-invaded | 11 | 0 | 0 | 0 | 6 | 0 |
|  | *C. purpureus* | 8 | 0 | 3 | 0 | 4 | 0 |
| END4 | Non-invaded | 6 | 0 | 9 | 0 | 1 | 0 |
|  | *C. purpureus* | 5 | 0 | 114 | 2 | 1 | 1 |
| END5 | Non-invaded | 16 | 0 | 8 | 0 | 3 | 1 |
|  | *C. purpureus* | 13 | 0 | 11 | 0 | 6 | 0 |
| END6 | Non-invaded | 36 | 0 | 6 | 0 | 7 | 0 |
|  | *C. purpureus* | 15 | 6 | 0 | 0 | 4 | 0 |
| END7 | Non-invaded | 3 | 0 | 0 | 0 | 2 | 0 |
|  | *H. coronarium* | 4 | 10 | 0 | 0 | 1 | 0 |
| END8 | Non-invaded | 0 | 0 | 13 | 0 | 1 | 0 |
|  | *M. maximus* | 0 | 0 | 0 | 2 | 0 | 0 |

Table S2. Number of larvae (larval clusters) and individual nymphs and adults of *Amblyomma sculptum* and *Amblyomma dubitatum* per vegetation types (non-invaded sites, *Hedychium coronarium*, *Cenchrus purpureus* and *Megathyrsus maximus*) from eight human-modified landscape areas (NEND 1 to NEND8) not endemic for Brazilian spotted fever in the state of São Paulo, Brazil.

| Areas | Vegetation type | Number of collected ticks | | | | | |
| --- | --- | --- | --- | --- | --- | --- | --- |
|  |  | *A.sculptum* larvae | *A.dubitatum* larvae | *A.sculptum* nymph | *A.dubitatum* nymph | *A.sculptum* adult | *A.dubitatum* adult |
| NEND1 | Non-invaded | 2 | 2 | 4 | 0 | 0 | 0 |
|  | *M. maximus* | 3 | 3 | 0 | 0 | 0 | 0 |
|  | *H. coronarium* | 11 | 0 | 0 | 0 | 0 | 0 |
| NEND2 | Non-invaded | 0 | 0 | 54 | 2 | 0 | 0 |
|  | *M. maximus* | 0 | 0 | 4 | 2 | 0 | 0 |
| NEND3 | Non-invaded | 1 | 3 | 0 | 0 | 0 | 0 |
|  | *C. purpureus* | 5 | 0 | 0 | 1 | 0 | 0 |
|  | *H. coronarium* | 1 | 0 | 1 | 0 | 0 | 0 |
| NEND4 | Non-invaded | 16 | 0 | 3 | 0 | 3 | 0 |
|  | *H. coronarium* | 16 | 0 | 7 | 0 | 0 | 0 |
| NEND5 | Non-invaded | 4 | 1 | 0 | 0 | 0 | 0 |
|  | *C. purpureus* | 7 | 0 | 1 | 1 | 1 | 0 |
|  | *H. coronarium* | 1 | 0 | 0 | 0 | 0 | 0 |
| NEND6 | Non-invaded | 4 | 0 | 1 | 3 | 0 | 0 |
|  | *H. coronarium* | 0 | 0 | 0 | 11 | 0 | 0 |
| NEND7 | Non-invaded | 0 | 2 | 6 | 0 | 0 | 0 |
|  | *M. maximus* | 0 | 0 | 0 | 0 | 0 | 0 |
|  | *H. coronarium* | 0 | 1 | 5 | 0 | 0 | 0 |
| NEND8 | Non-invaded | 0 | 0 | 32 | 1 | 0 | 0 |
|  | *C. purpureus* | 7 | 8 | 30 | 0 | 0 | 0 |
|  | *H. coronarium* | 0 | 0 | 4 | 1 | 0 | 0 |

Table S3. Number of larvae (larval clusters) and individual nymphs and adults of *Amblyomma sculptum* and *Amblyomma dubitatum* per vegetation types (non-invaded sites, *Hedychium coronarium*, *Cenchrus purpureus* and *Megathyrsus maximus*) from eight natural reserve areas (UC1 to UC8) within conservation units of the Atlantic Rainforest biome in the state of São Paulo, Brazil.

| Areas | Vegetation | *A.sculptum* larvae | *A.dubitatum* larvae | *A.sculptum* nymph | *A.dubitatum* nymph | *A.sculptum* adult | *A.dubitatum* adult |
| --- | --- | --- | --- | --- | --- | --- | --- |
| UC1 | Non-invaded | 3 | 0 | 8 | 4 | 5 | 0 |
|  | *M. maximus* | 1 | 0 | 0 | 5 | 0 | 0 |
| UC2 | Non-invaded | 4 | 0 | 0 | 0 | 0 | 0 |
|  | *M. maximus* | 2 | 1 | 10 | 2 | 0 | 0 |
| UC3 | Non-invaded | 0 | 0 | 5 | 2 | 0 | 0 |
|  | *M. maximus* | 4 | 0 | 0 | 0 | 0 | 0 |
| UC4 | Non-invaded | 0 | 0 | 0 | 0 | 0 | 0 |
|  | *C. purpureus* | 1 | 0 | 0 | 0 | 0 | 0 |
|  | *H. coronarium* | 0 | 1 | 0 | 3 | 0 | 0 |
| UC5 | Non-invaded | 0 | 0 | 0 | 2 | 0 | 0 |
|  | *H. coronarium* | 0 | 6 | 0 | 6 | 0 | 1 |
| UC6 | Non-invaded | 0 | 3 | 0 | 1 | 0 | 1 |
|  | *H. coronarium* | 0 | 3 | 0 | 1 | 0 | 0 |
| UC7 | Non-invaded | 1 | 3 | 0 | 0 | 0 | 0 |
|  | *M. maximus/C. purpureus* | 0 | 7 | 0 | 0 | 0 | 0 |
| UC8 | Non-invaded | 0 | 1 | 0 | 0 | 0 | 0 |
|  | *M. maximus* | 2 | 0 | 6 | 3 | 0 | 0 |
|  | *H. coronarium* | 0 | 0 | 6 | 0 | 0 | 0 |

Table S4. Numerical data of the density of larvae, nymphs and adults of *Amblyomma sculptum* and *Amblyomma dubitatum* in the three vegetation types (H.cor: *Hedychium coronarium*; C.p/M.m: *Cenchrus purpureus* and *Megathyrsus maximus*; N.i.s.: non-invaded sites) within the three types of areas (END, NEND and UC) sampled in the present study. In each vegetation type of each area, the total number of larval clusters, individual nymphs and adults were divided by the total area covered by flannel dragging to calculate the number of ticks per square meter (ticks/m^2^).

| Tick stage | Tick density: number of ticks per m^2^ according to the area (END, NEND, UC), tick species, and vegetation type (H.cor, C.p/M.m, N.i.s.) | | | | | | | | | | | | | | | | | | | | | | |
| --- | --- | --- | --- | --- | --- | --- | --- | --- | --- | --- | --- | --- | --- | --- | --- | --- | --- | --- | --- | --- | --- | --- | --- |
|  | Endemic areas (END) | | | | | | |  | Non-endemic areas (NEND) | | | | | | |  | Conservation units (UC) | | | | | | |
|  | *A. sculptum* | | |  | *A. dubitatum* | | |  | *A. sculptum* | | |  | *A. dubitatum* | | |  | *A. sculptum* | | |  | *A. dubitatum* | | |
|  | H.cor | C.p/M.m | N.i.s. |  | H.cor | C.p/M.m | N.i.s. |  | H.cor | C.p/M.m | N.i.s. |  | H.cor | C.p/M.m | N.i.s. |  | H.cor | C.p/M.m | N.i.s. |  | H.cor | C.p/M.m | N.i.s. |
|  |  |  |  |  |  |  |  |  |  |  |  |  |  |  |  |  |  |  |  |  |  |  |  |
| Larva* | 0.040 | 0.078 | 0.122 |  | 0.093 | 0.011 | 0.000 |  | 0.055 | 0.049 | 0.045 |  | 0.002 | 0.024 | 0.013 |  | 0.000 | 0.022 | 0.013 |  | 0.033 | 0.018 | 0.012 |
|  |  |  |  |  |  |  |  |  |  |  |  |  |  |  |  |  |  |  |  |  |  |  |  |
| Nymph | 0.000 | 0.459 | 0.080 |  | 0.000 | 0.013 | 0.000 |  | 0.032 | 0.078 | 0.167 |  | 0.023 | 0.009 | 0.010 |  | 0.020 | 0.036 | 0.022 |  | 0.033 | 0.022 | 0.015 |
|  |  |  |  |  |  |  |  |  |  |  |  |  |  |  |  |  |  |  |  |  |  |  |  |
| Adult | 0.047 | 0.040 | 0.047 |  | 0.000 | 0.002 | 0.002 |  | 0.000 | 0.002 | 0.005 |  | 0.000 | 0.000 | 0.000 |  | 0.000 | 0.000 | 0.008 |  | 0.003 | 0.000 | 0.002 |
|  |  |  |  |  |  |  |  |  |  |  |  |  |  |  |  |  |  |  |  |  |  |  |  |

* refer to the number of larval clusters

Table S5. Results of the molecular identification of pools of *Amblyomma* spp. larvae that were collected on different vegetation types in different conservation unit areas (UC1 to UC6, UC8) of the Atlantic Rainforest biome in the state of São Paulo, Brazil.

| Area | Vegetation Type | Closest identity in GenBank: % to tick species (accession number) | GenBank accession number of the generated sequence |
| --- | --- | --- | --- |
| UC1 | *Megathyrsus maximus* | 100% to *A. sculptum* (MK059460) | PX435257 |
| UC2 | *M. maximus* | 100% to *A. sculptum* (MK059460) | PX435258 |
| UC2 | *M. maximus* | 100% to *A. sculptum* (MK059460) | PX435259 |
| UC2 | Non-invaded site | 100% to *A. sculptum* (MK059460) | PX435260 |
| UC3 | *M. maximus* | 100% to *A. sculptum* (MK059460) | PX435261 |
| UC3 | *M. maximus* | 100% to *A. sculptum* (MK059460) | PX435262 |
| UC4 | *Cenchrus purpureus* | 100% to *A. sculptum* (KY172626) | PX435263 |
| UC5 | *Hedychium coronarium* | 100% to *A. dubitatum* (MT275640) | PX435264 |
| UC5 | *H. coronarium* | 100% to *A. dubitatum* (MT275640) | PX435265 |
| UC6 | *H. coronarium* | 100% to *A. dubitatum* (MT275640) | PX435266 |
| UC6 | Non-invaded site | 100% to *A. dubitatum* (MT275640) | PX435267 |
| UC8 | *M. maximus* | 99.7% to *A. sculptum* (KY172626) | PX435268 |
