# Supplementary material for: Assessment of tick populations associated with capybaras in natural reserves and human-modified environments with or without invasive plants in the state of São Paulo, Brazil
Source: Exp Appl Acarol. 2026 Mar 14;96(3):34. doi: 10.1007/s10493-026-01127-w (PMC12988901; doi:10.1007/s10493-026-01127-w)
Supplement: Supplementary file 3 — Supplementary Material 3 [file 10493_2026_1127_MOESM3_ESM.pdf]

Supplementary Data S1

Model results: Tick density - **LARVAE**

VARIABLE	ESTIMATE	CI SUPERIOR (ESTIMATE)	CI INFERIOR (ESTIMATE)	STD. ERROR	P-VALUE	ODDS RATIO	CI INFERIOR (IRR)	CI SUPERIOR (IRR)	PERCENTAGE CHANGE (%)
**INTERCEPT**	-4.70476	-3.6807972	-5.7287228	0.52243	**< 2e-16**	0.009	0.00325122705	0.02520287509	-99.09479135
**HEDICHIUM**	1.47726	2.580446	0.374074	0.56285	**0.00867**	4.38	1.453644716	13.2030254	338.0925485
**GRASS**	0.75641	1.868612	-0.355792	0.56745	0.18253	2.13	0.7006183337	6.479296891	113.0613572
**SCULPTUM**	0.39452	1.8149516	-1.0259116	0.72471	0.58618	1.48	0.3584695356	6.140778957	48.36718575
**NEND AREA**	-0.17109	0.9049304	-1.2471104	0.54899	0.7553	0.84	0.2873338784	2.471759883	-15.72542771
**END AREA**	-0.02912	1.061424	-1.119664	0.5564	0.95826	0.97	0.3263894431	2.890484109	-2.870009852
**SCULPTUM: NEND**	1.75143	3.2767216	0.2261384	0.77821	**0.02441**	5.76	1.253749172	26.48878935	476.2837644
**SCULPTUM: END**	1.96372	3.5030256	0.4244144	0.78536	**0.01241**	7.12	1.528694954	33.2157978	612.5785744
**HEDICHIUM: SCULPTUM**	-2.1268	-0.596334	-3.657266	0.78085	**0.00645**	0.11	0.02580296168	0.550827272	-88.07818177
**GRASS: SCULPTUM**	-0.73298	0.755542	-2.221502	0.75945	0.33447	0.48	0.1084461004	2.128765001	-51.95249611

In the model, we observe that:

● The density of *A. dubitatum* increases significantly in HEDICHIUM plots compared to the reference plots Non-invaded sites (NIS). No difference was found between GRASS pasture and NIS for this species.

A. dubitatum in HEDICHIUM plots has a density 4.38 times (95% CI: 1.45-13.38; p = 0.0087) higher than in the reference plots (NIS).

● The density of *A. sculptum* decreases significantly in HEDICHIUM plots compared to the reference plots (NIS). No difference was found between GRASS pasture and NIS for this species.

The density of *A. sculptum* in the HEDICHIUM plots decreased by approximately 88% (IRR = 0.119; 95% CI: 0.02-0.61; p = 0.006) compared to the reference plots (NIS).

● The density of *A. dubitatum* does not appear to vary according to the type of area (UC, END, NEND).

● The density of *A. sculptum* increases significantly in both END and NEND areas compared to the reference category (UC).

A. sculptum in the NEND area has a density 5.76 times (95% CI: 1.25-25.89; p = 0.02) higher than in the reference area (UC).

The density of *A. sculptum* in the END area is 7.12 times higher (95% CI: 1.52-33.04; p = 0.02) than in the reference area (UC).

● Although the species A. sculptum has higher densities than A. dubitatum, this difference is not significant when the model includes the other variables.

Model results: Tick density - **NYMPHS**

VARIABLE	ESTIMATE	CI INFERIOR (ESTIMATE)	CI SUPERIOR (ESTIMATE)	STD. ERROR	P-VALUE	ODDS RATIO	CI INFERIOR (IRR)	CI SUPERIOR (IRR)	PERCENTAGE CHANGE (%)
**INTERCEPT**	-4.3758	-5.327968	-3.423632	0.4858	**< 2e-16**	0.01	0.00485392317	0.03259383888	-98.74219242
**HEDICHIUM**	0.9048	-0.220828	2.030428	0.5743	0.115188	2.47	0.8018545874	7.617345885	147.1437586
**GRASS**	0.8321	-0.252172	1.916372	0.5532	0.13258	2.29	0.7771110635	6.796256868	129.813977
**SCULPTUM**	0.8457	-0.416148	2.107548	0.6438	0.188997	2.32	0.659582645	8.228041372	132.960797
**NEND AREA**	-0.5052	-1.49108	0.48068	0.503	0.315243	0.6	0.2251293845	1.617173707	-39.66151136
**END AREA**	-1.5146	-2.73568	-0.29352	0.623	**0.01505**	0.21	0.06484989418	0.74563431	-78.01038743
**SCULPTUM: NEND**	1.7864	0.439488	3.133312	0.6872	**0.009335**	5.96	1.551912436	22.94986376	496.7929203
**SCULPTUM: END**	2.9503	1.415424	4.485176	0.7831	**0.000165**	19.1	4.118232226	88.69255933	1811.168637
**HEDICHIUM: SCULPTUM**	-2.141	-3.620212	-0.661788	0.7547	**0.004553**	0.11	0.02677699917	0.51592803	-88.2462753
**GRASS: SCULPTUM**	-0.1786	-1.552756	1.195556	0.7011	0.798946	0.83	0.2116638237	3.305395059	-16.35595913

In the model, we observe that:

● Nymphs of *A. dubitatum* show lower density in END areas.

A. dubitatum in the END area shows a reduction of approximately 78% (IRR = 0.21; 95% CI: 0.06-0.7; p = 0.001) in density compared to the reference area (UC).

● Nymphs of *A. dubitatum* show no difference in density compared to GRASS.

● *A. sculptum* shows lower densities in HEDICHIUM, repeating what was found in LARVAE.

A. sculptum in HEDICHIUM plots shows a reduction of approximately 88% (IRR = 0.11; 95% CI: 0.02-0.75; p = 0.004) in density compared to the reference area (UC).

● The density of *A. sculptum* nymphs increases in END and NEND areas compared to UC.

A. sculptum in the END area has a density 19.1 times (95% CI: 4.11-88.69; p = 0.0001) higher than in the reference area (UC).

A. sculptum in the NEND area has a density 5.96 times (95% CI: 1.55-22.95; p = 0.009) higher than in the reference area (UC).

Model results: Tick density – **ADULTS**

VARIABLE	ESTIMATE	CI INFERIOR (ESTIMATE)	CI SUPERIOR (ESTIMATE)	STD. ERROR	P-VALUE	ODDS RATIO	CI INFERIOR (IRR)	CI SUPERIOR (IRR)	PERCENTAGE CHANGE (%)
**INTERCEPT**	-6.465	-8.208812	-4.721188	0.8897	**3.69e-13**	0.001	0.0002722439551	0.008904593605	-99.84430087
**HEDICHIUM**	0.3099	-2.11462	2.73442	1.237	0.8022	1.36	0.1206791389	15.40080838	36.32887784
**GRASS**	-0.4906	-2.8916	1.9104	1.225	0.6889	0.61	0.05548736177	6.755790574	-38.77410714
**SCULPTUM**	1.059	-0.90688	3.02488	1.003	0.2908	2.88	0.4037820608	20.59153358	188.3486061
**NEND AREA**	-0.3626	-0.3626007595	-0.3625992405	0.0000003875	1	0.69	0.6958641952	0.6958652522	-30.41352763
**END AREA**	0.1318	-1.85368	2.11728	1.013	0.8965	1.14	0.1566595969	8.308507584	14.08801204
**SCULPTUM: NEND**	0.3591	0.3590992454	0.3591007546	0.000000385	1	1.43	1.432038918	1.432041079	43.20399984
**SCULPTUM: END**	2.324	0.13664	4.51136	1.116	**0.0373**	10.2	1.146415365	91.04555631	921.6458517
**HEDICHIUM: SCULPTUM**	-0.7496	-3.3074	1.8082	1.305	0.5658	0.47	0.03661123934	6.09945852	-52.74444628
**GRASS: SCULPTUM**	0.15	-2.3098	2.6098	1.255	0.9049	1.16	0.0992811058	13.59633131	16.18342427

In the model, we observe that:

● No significant difference in rates was observed when changing the type of vegetation.

● The density of *A. sculptum* is higher in END areas when compared to the reference category.

A. *sculptum* in the END area has a density 10.2 times (95% CI: 1.14-91.04; p = 0.03) higher than in the reference area (UC).
